# Short and Efficient Synthesis of the Antituberculosis Agent Pretomanid from (*R*)-Glycidol

**DOI:** 10.1021/acs.oprd.3c00187

**Published:** 2023-09-05

**Authors:** Tobias Lucas, Jule-Philipp Dietz, Flavio S. P. Cardoso, David R. Snead, Ryan C. Nelson, Kai O. Donsbach, B. Frank Gupton, Till Opatz

**Affiliations:** † Department of Chemistry, Johannes Gutenberg-University, Duesbergweg 10−14, 55128 Mainz, Germany; ‡ Department of Chemical and Life Sciences Engineering, Virginia Commonwealth University, Richmond, Virginia 23284, United States

**Keywords:** pretomanid, tuberculosis, nitroimidazoles, active pharmaceutical ingredient, cyclization

## Abstract

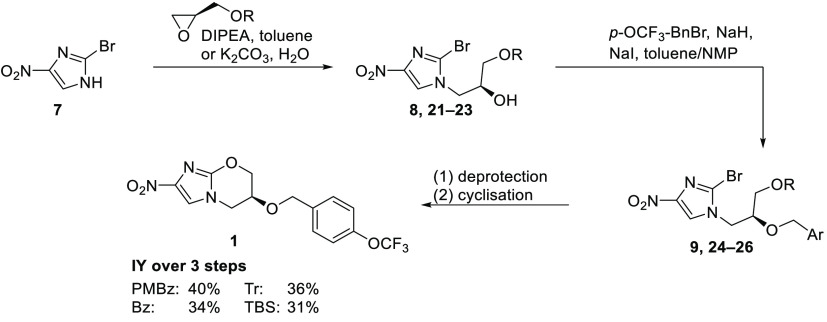

An efficient gram-scale synthesis of the antituberculosis agent pretomanid using straightforward chemistry, mild reaction conditions, and readily available starting materials is reported. Four different protecting groups on the glycidol moiety were investigated for their technical feasibility and ability to suppress side reactions. Starting from readily available protected (*R*)-glycidols and 2-bromo-4-nitro-1*H*-imidazole, pretomanid could be prepared in a linear three-step synthesis in up to 40% isolated yield. In contrast to most syntheses reported so far, deprotection and cyclization were performed in a one-pot fashion without any hazardous steps or starting materials.

## 1 Introduction

With an enormous infection prevalence of around one-quarter of the entire global human population, tuberculosis (TB), commonly caused by *Mycobacterium tuberculosis*, is one of the most significant health threats of our age. Although most of the infections remain latent, TB is still one of the leading causes of death through infection worldwide.^1^ Compared to other infectious diseases, TB currently has a high morbidity and mortality (in 2020, 10 million people contracted active TB and 1.8 million people died from the disease).^2,3^ Particularly in combination with the AIDS pandemic, it is still one of the major causes of death in Africa.^4,5^

**Figure 1:**
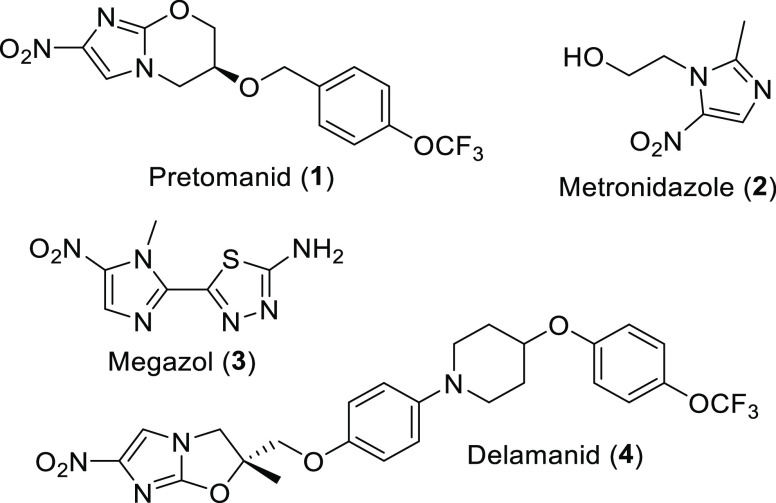
Structures of pretomanid (**1**), metronidazole (**2**), megazol (**3**), and delamanid (**4**), which compromise the nitroimidazole class of APIs.

Pretomanid (**1**) belongs to the class of nitroimidazoles which dates back to the 1960s and enjoys a revived attention in recent years. The antibiotic and antiprotozoal drug metronidazole (**2**), one of the earliest representatives of this drug class, is still in use as an important medication and is on the WHO’s “List of Essential Medicines”.^6^ It can be used as an antibiotic and as an antiprotozoal medication. As diseases caused by protozoans remain a considerable problem in low-income countries, nitroimidazoles like metronidazole (**2**) and megazol (**3**) are highly important medicines. Besides pretomanid (**1**), the related compound delamanid (**4**) belongs to antituberculosis agents that are highly effective against multiresistant strains (Figure 1).^7^

**Figure 2:**
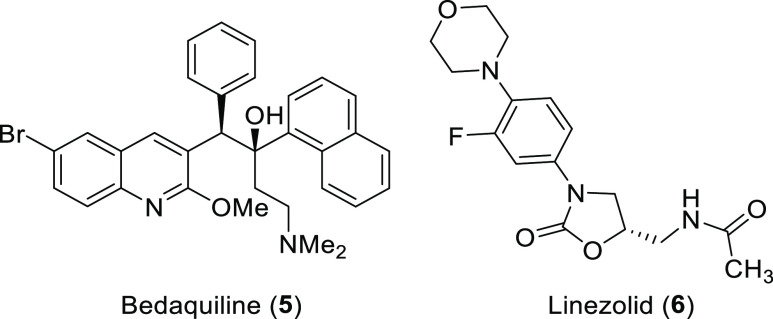
Structures of bedaquiline (**5**) and linezolid used (**6**) in multidrug therapy together with pretomanid (**1**) (BPaL).

Pretomanid (**1**), first described in 1997 by Barry and Baker (Pathogenesis Corp.),^8^ belongs to a novel class of antituberculosis agents.^9^ It was approved by the FDA in 2019^10^ and was subsequently recommended by the World Health Organization (WHO) for the treatment of multidrug-resistant (MDR) tuberculosis^11,12^ and extensively resistant (XDR)^13,14^ tuberculosis in combination with bedaquiline (**5**)^7,15^ and linezolid (**6**) as part of a multidrug therapy (BPaL) (Figure 2).^16−18^

Several synthetic pathways to pretomanid (**1**) have been investigated so far.^8,19−24^ The first route was reported by Barry and Baker (Pathogenesis Corp.) in 1997.^8^ Starting from the explosive 2,4-dinitroimidazole, pretomanid (**1**) was prepared in five steps and 17% overall yield. The synthesis also includes other potentially hazardous or undesirable conditions like the use of NaH/DMF, which can be problematic for scale-up in a technical process due to the associated explosion risk.^25^ In 2013, Read and Fairlamb^19^ reported a modified version of the initial route. Their synthesis starts with the safe and readily available 2-bromo-4-nitroimidazole (**7**), which undergoes a nucleophilic substitution with a TBS-protected glycidol. This is followed by installation of the aryl moiety, cleavage of the protecting group, and final cyclization to **1**. Over four steps, pretomanid (**1**) was obtained in 10% yield, and the sequence contained multiple chromatographic purifications.

**Scheme 1:**
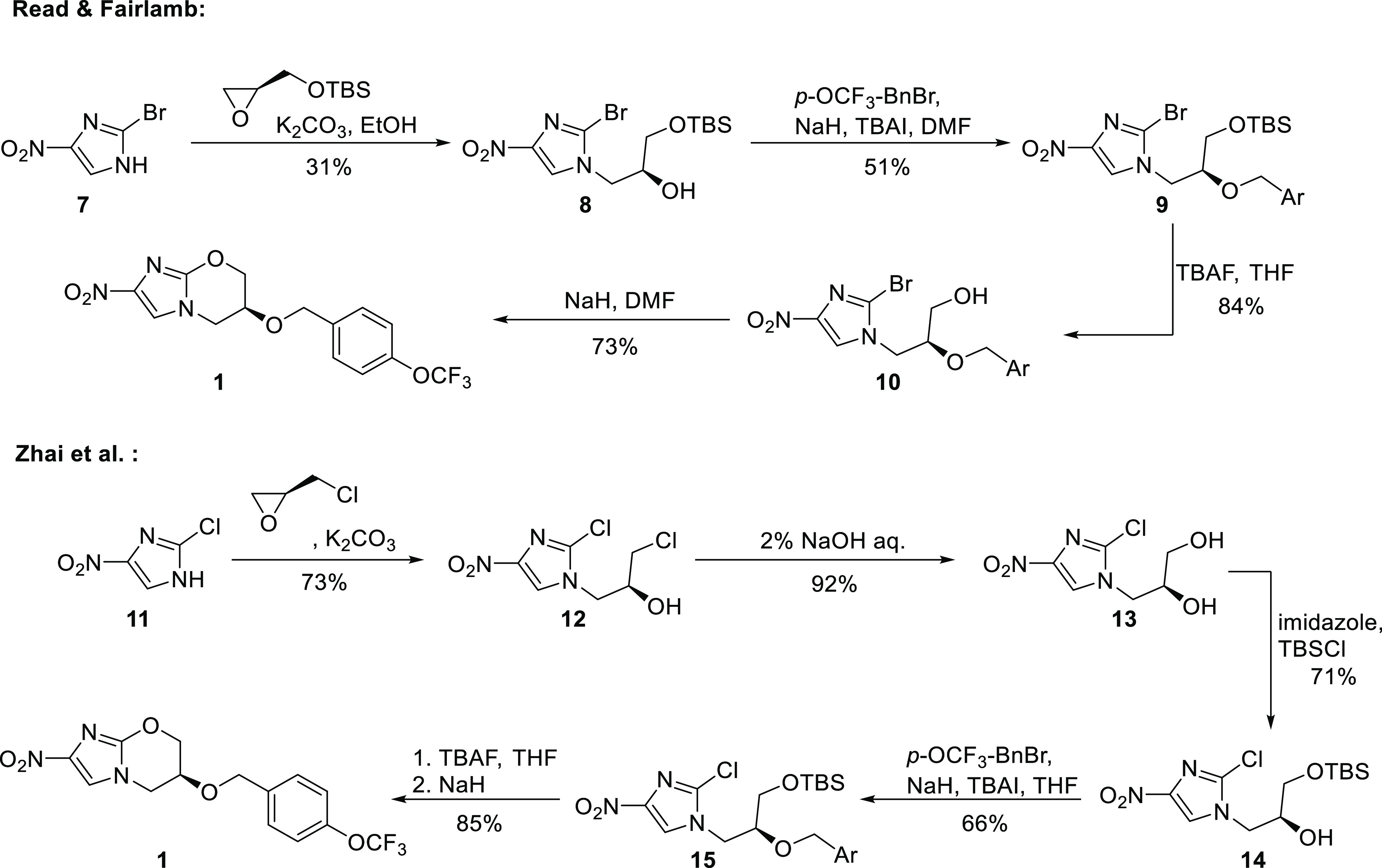
Synthesis of Pretomanid (**1**) by Read and Fairlamb^19^ and Zhai et al.^20^

In 2020, Zhai et al.^20^ reported a different approach to pretomanid (**1**), starting from 2-chloro-4-nitroimidazole (**11**) and (*S*)-epichlorohydrin (Scheme 1). Imidazole **11** was first *N*-alkylated, followed by hydrolysis to diol **13**. Selective TBS protection of the primary alcohol enabled the benzylation of the secondary hydroxyl group to furnish alcohol **14**. Subsequent one-pot cleavage of the silyl-protecting group and cyclization afforded pretomanid (**1**) in 28% overall yield.

**Scheme 2:**
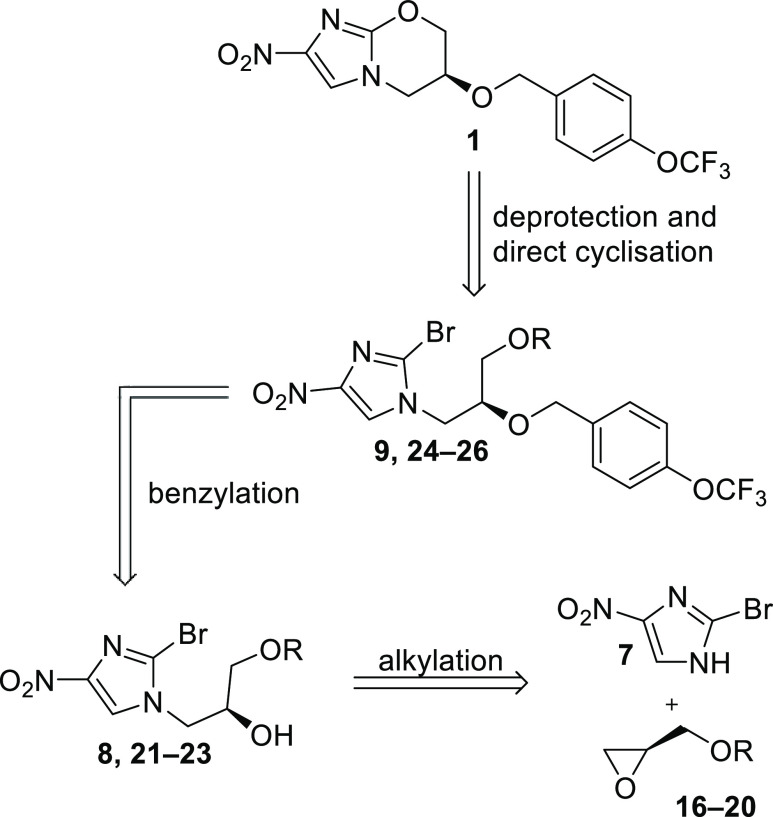
Retrosynthetic Synthesis Plan to Pretomanid (**1**)

Herein, an optimized synthetic route is reported, starting from readily accessible protected (*R*)-glycidols (**16–20**) and nitroimidazole **7**. After alkylation of **7** through epoxide opening and *O*-alkylation of the resulting secondary alcohol, a one-pot deprotection/cyclization to pretomanid (**1**) was envisioned (Scheme 2). Different protecting groups were tested preemptive to reduce the potential migration of the protecting group, which results in the undesired regioisomer in the *O*-benzylation. Another key aspect of this work was to establish an easily applicable, technically feasible, and scalable synthesis without the need for chromatographic steps.

## 2 Results and Discussion

### 2.1 Preparation of 2-Bromo-4-nitro-1*H*-imidazole (**7**)

The published process for preparing the starting material 2-bromo-4-nitroimidazoline (**7**)^26^ revealed optimization potential with respect to process safety and isolated yield. The use of molecular bromine, difficult on a larger scale, could be replaced with the in situ formation of bromine from hydrobromic acid and hydrogen peroxide.

**Scheme 3:**
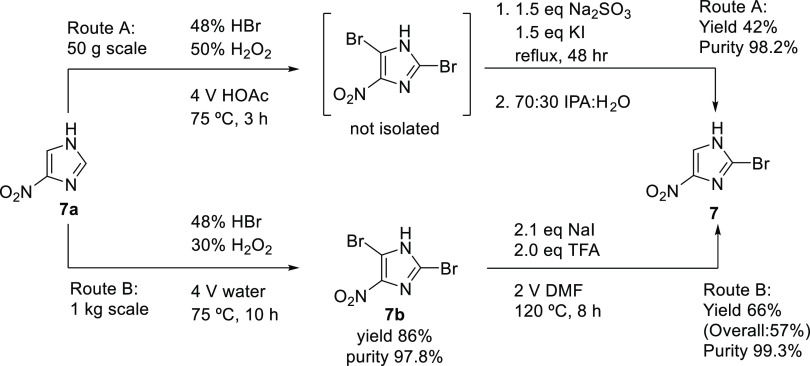
Preparation of Starting Material 2-Bromo-4-nitroimidazoline (**7**)

The intermediate 2,5-dibromo-4-nitroimidazole does not require isolation but could be debrominated in a single pot to yield **7** in moderate yields (Scheme 3A). However, on scaling this one-pot procedure, a decrease in product purity was observed due to intractable salt contamination, so the final procedure run on kilogram scale involved isolation of **7b** and debromination with NaI and TFA to yield 2-bromo-4-nitroimidazoline (**7**) in good yields and very high purity (Scheme 3B). Although the conditions described herein have been run on a 1–2 kg scale, the authors wish to point out that further safety studies will be required to scale this reaction further.^27^

### 2.2 Preparation of Protected (*R*)-Glycidols

Preparation of the protected (*R*)-glycidol derivatives **16**–**20** was performed according to literature procedures (Table 1). Glycidols **16**, **17**, and **19** were purified by distillation, whereas compounds **18** and **20** were used for the next step without prior purification.

**Table 1:**
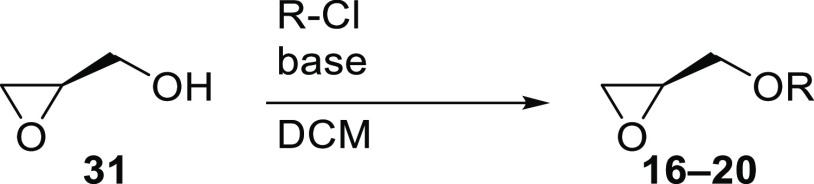
*O*-Protection of (*R*)-Glycidol (**31**)

entry	R-Cl (equiv)	IY [%]	ref
1	PMBzCl (4-anisoyl chloride)	82 (**16**)^c^	(28)
2	BzCl	84 (**17**)^c^	(29)
3	TrCl	94 (**18**)	(30)
4	TBSCl	99 (**19**)^c^	(31)
5	TMSCl	76 (**20**)	(30)

a 0.01 equiv DMAP was added.

b 0.05 equiv DMAP was added.

c After distillation.

### 2.3 Alkylation of 2-Bromo-4-nitro-1*H*-imidazole (7)

The alkylation of imidazole **7** using the *p-*anisoyl-protected PMBz-(*R*)-glycidol **16** was carried out in toluene at slightly elevated temperatures using DIPEA as a base (Table 2, entry 1). The desired product **21** precipitates during the reaction, causing a beige suspension after 48 h. Filtration and washing with toluene were insufficient as a significant amount of product was lost in the mother liquor. Therefore, toluene was removed prior to redissolving the residue in ethyl acetate to obtain a homogeneous solution. HPLC data from this solution revealed 99% conversion of imidazole **7** to produce 79% of the desired isomer **21** (HPLC, 315 nm). Observed side products were assumed to be the *N′-* and *O*-regioisomers as well as cyclized compounds on the basis of HPLC-MS data (see the Supporting Information for more details). After washing the ethyl acetate phase with aqueous HCl and NaHCO_3_ solution, crude **21** was obtained as a yellowish-orange solid showing a purity of 83% (HPLC, 315 nm). Purification attempts by precipitation from ethyl acetate/*n*-heptane only led to a minor increase in purity (86%). Due to a loss of product, and in order to achieve a high overall yield, crude **21** was used for further transformations.

**Table 2:**
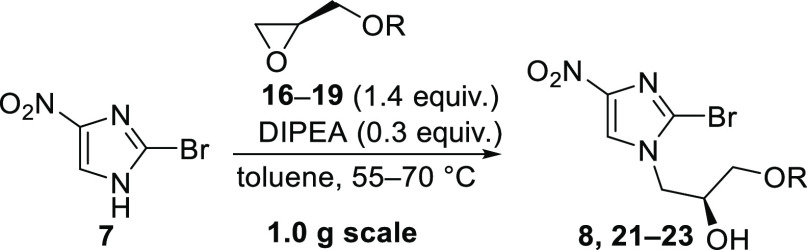
Alkylation of 2-Bromo-4-nitro-1*H*-imidazole (**7**) Using *O*-Protected (*R*)-Glycidols **8** and **21**–**23** in Toluene/DIPEA

entry	R	*T* [°C]	*t* [h]	conversion [%]^a^	area [%]^a^	IY [%]^b^	purity^a^,^c^ [%]
1	PMBz (**21**)	55	48	99	79	>100	83
2	Bz (**22**)	57	45	99	81	>100	87
3	Tr (**23**)	70	46	98	81	>100	82
4	TBS (**8**)	70	72	99	85	>100	86

a Determined by HPLC (area % at 315 nm).

b Crude yield.

c After workup.

When using Bz-glycidol **17** (Table 2, entry 2) for the alkylation, similar results in terms of conversion were observed. In contrast to the crystalline PMBz-derivative **21**, crude imidazole **22** was however isolated as a viscous oil (HPLC purity 81%, 315 nm). Adding MTBE (methyl *tert*-butyl ether) to the solution of crude **22** in ethyl acetate led to precipitation of contaminants which increased the purity of imidazole **22** remaining in the organic layer to 87% (HPLC, 315 nm).

Trityl-glycidol **18** (Table 2, entry 3) was found to be less reactive in the alkylation of imidazole **7**. Thus, the temperature had to be increased to 70 °C, which gave a 98% consumption of **18** (81% conversion to **23**, HPLC, 315 nm) after 46 h. Crude imidazole **23** was obtained as a brownish solid and was used in the next step without further purification.

Crude TBS-protected nitroimidazole **8** (Table 2, entry 4) was obtained as an orange viscous oil under similar conditions as the Bz-derivative. In contrast to the other protecting groups tested here, most of the impurities could be separated from the desired product **8** by recrystallization. However, the use of crude **8** for the following steps proved superior in terms of the final yield. Alkylation with the TMS-protected glycidol **20** did not give the desired product and only side product formation was observed instead.

Further investigations revealed that the alkylation of **7** could also be performed in water using K_2_CO_3_ as a base. In the case of PMBz-glycidol **21**, similar conversions as with toluene/DIPEA were observed (Table 3, entry 1). Crude alcohol **21** also precipitated out during the reaction. As only traces of **21** could be detected in the liquid fraction of the reaction mixture, the product was isolated by filtration. Washing the solid with water furnished crude **21** of improved HPLC purity (87%), which could be further increased to 98% by precipitation from ethyl acetate/*n*-heptane.

**Table 3:**
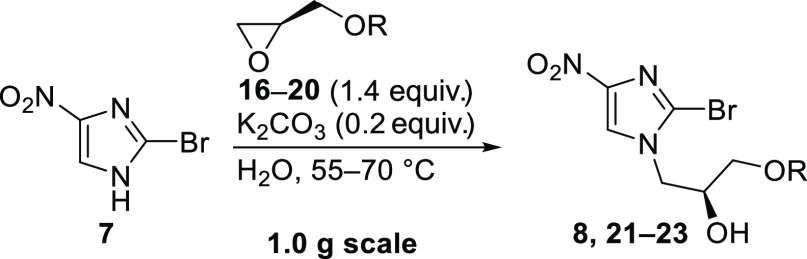
Alkylation of 2-Bromo-4-nitroimidazole (**7**) Using *O*-Protected (*R*)-Glycidols **16**–**19** in Water/K_2_CO_3_

entry	R	*T* [°C]	*t* [h]	conversion [%]^a^	area [%]^a^	IY [%]^b^	purity [%]^a^
1	PMBz (**21**)	55	44	94	78	>100	87
2	Bz (**22**)	55	71	97	74	>100	81
3	Tr (**23**)	70	72				
4	TBS (**8**)	65	72		85	50	

a Determined by HPLC (area % at 315 nm).

b Crude yield.

When using Bz-glycidol (Table 3 entry 2), crude alcohol **22** precipitated as a colorless sticky solid. HPLC data from the ethyl acetate extract revealed a 97% consumption of **17** (74% conversion to **22**, 315 nm). After washing with aq. HCl and NaHCO_3_-solution, the purity of crude **22** was slightly improved (81%, 315 nm).

Trityl-glycidol **18** as well as TBS-glycidol **19** were not suitable for the alkylation reaction in water (Table 3, entries 3 and 4) due to their poor solubility. For trityl-glycidol **18**, a high temperature (70 °C) had to be applied to observe conversion. Mainly side product formation was detected under these conditions, presumably due to undesired hydrolysis of the epoxide ring. Similar observations were made with TBS-glycidol **19**.

### 2.4 Alkylation of the Secondary Alcohol Using 4-(Trifluoromethoxy)benzyl Bromide

For the *O*-alkylation step, the purity of crude alkylated imidazoles **8**, **21**–**23** was sufficient, and it was possible to use them without cumbersome purification steps which also prevented a loss of overall yield (Table 4). A solution of alkylated imidazole **8**, **21**–**23** in NMP was added slowly over 2 h to a suspension of 4-(trifluoromethoxy)benzyl bromide and NaH in toluene/NMP. Careful addition and temperature control were crucial to suppress undesired cyclized side products. In general, the reaction proceeded with high overall conversion (96–99%, HPLC, 315 nm) of alkylated imidazoles **8**, **21**–**23**, followed by quenching the reaction mixture with acetic acid/water and extraction with toluene to furnish the benzylated products (75–90%, HPLC, 315 nm).

**Table 4:**
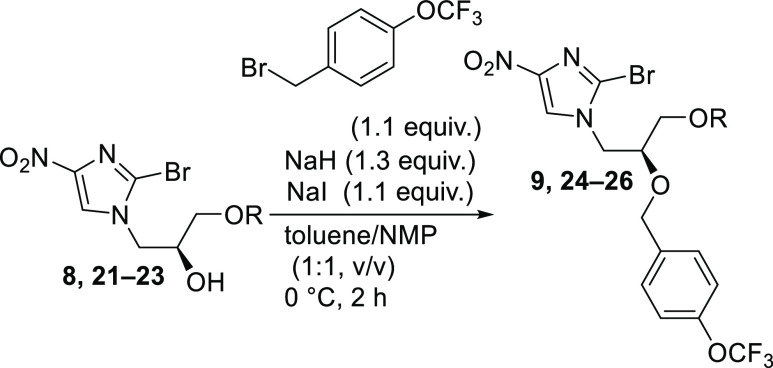
Alkylation of the Secondary Hydroxy Group

entry	R	conv. [%]^a^	area **9**, **24**–**26** [%]^a^	IY [%]	purity [%]
1	PMBz	96	82	>100	82
2^b^	PMBz	96	81	>100	81
3	Bz	97	82	>100	84
4^b^	Bz	96	75	>100	74
5	Tr	99	79	>100	73
6	TBS	99	90	>100	90

a Determined by HPLC (area % at 315 nm).

b Derived from the alkylation performed in water.

### 2.5 Deprotection and Cyclization to Pretomanid

**Scheme 4:**
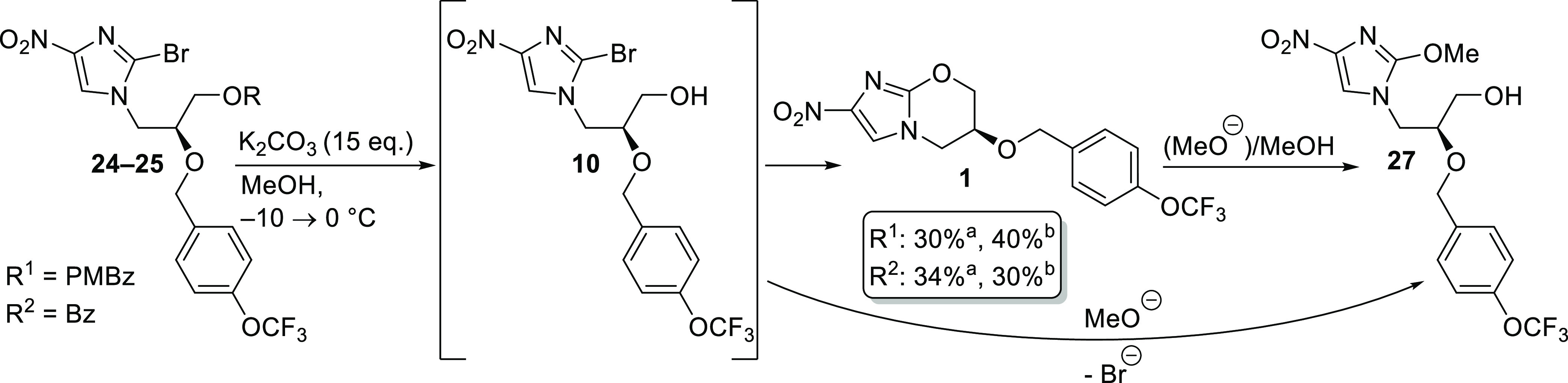
One-Pot Deprotection and Cyclization to Pretomanid (**1**) in the PMBz and Bz Route Isolated yield over three steps; alkylation performed in toluene/DIPEA. Isolated yield over three steps; alkylation performed in water/K_2_CO_3_.

A one-pot deprotection/cyclization reaction to pretomanid (**1**) was achieved using an excess of K_2_CO_3_ in MeOH in the case of PMBz- and Bz-protected starting materials **24**–**25**. It was important to perform the reaction at low temperatures and follow the conversion carefully by HPLC because **10** was observed to react with in situ generated methoxide to form side product **27** (Scheme 4, see the SI for more details). Moreover, pretomanid (**1**) was prone to ring-opening by methoxide at longer reaction times.

A methanolic solution of crude **24** or **25** was cooled to −10 °C and an excess of K_2_CO_3_ was added at once. After 2 h, HPLC data showed complete saponification to **10**. Keeping this temperature overnight led to the predominant formation of side product **27**. Warming to room temperature led to the same result. When the reaction mixture was warmed to 0 °C and kept overnight (14 h), only 10–12% of side product **27** was formed along with 60–70% of pretomanid **1**. For workup, the reaction was quenched by the addition of water. While stirring overnight at ambient temperature, crude pretomanid (**1**) precipitated. After filtration and drying, the solid was suspended in hot MTBE to remove impurities. Pretomanid (**1**) was obtained as a colorless solid (HPLC purity: ≥99%, 315 nm) in 30–40% overall isolated yield.

One of the major intentions of investigating the trityl and TBS routes was to replace methanol as required for the ester cleavage due to the risk of nucleophilic substitution of bromine in **7**. Crude imidazole **26** (80 area %, HPLC, 315 nm) was readily deprotected in the presence of HCl yielding crude alcohol **10** (79 area %, 315 nm).

Cyclization attempts using NaH in THF were unsuccessful. Only 21% consumption (12% conversion to **1**) could be observed when 3.0 equiv of NaH were added. Further addition of NaH led to a complex product mixture.

**Scheme 5:**
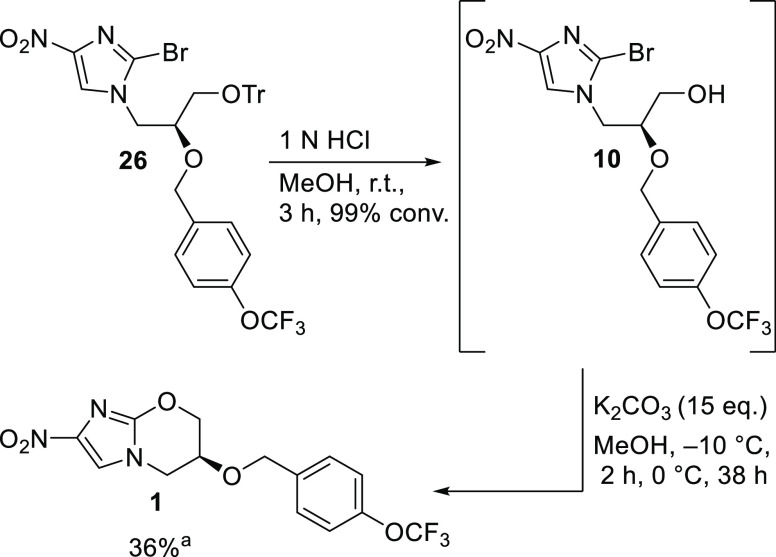
One-Pot Deprotection and Cyclization of Crude (*S*)-2-Bromo-4-nitro-1-(2-((4-(trifluoromethoxy)benzyl)oxy)-3-(trityloxy)propyl)-1*H*-imidazole (**26**) to Pretomanid (**1**) Isolated yield over three steps.

To use the conditions applied for the Bz and PMBz route (K_2_CO_3_ in MeOH), precursor **26** first had to be deprotected using methanolic HCl followed by K_2_CO_3_ addition at −10 °C. After stirring for 38 h at 0 °C, **10** was completely consumed yielding 50% pretomanid (**1**) as well as 11% of the methanol adduct **27**. After workup, pretomanid (**1**) was obtained in 36% overall yield (Scheme 5).

For the one-pot deprotection and cyclization of TBS-protected alcohol **9** to pretomanid (**1**), various conditions were investigated (Table 5). In order to replace MeOH, various solvents as well as different conditions for the deprotection of intermediate **10** and subsequent cyclization to **1** were tested. As in the trityl route, HCl in 1,4-dioxane was used to remove the TBS group. For the cyclization, potassium carbonate was used first, but side product formation was observed. By switching to sodium hydride, only the displacement of the primary hydroxy group by chloride was observed.

**Table 5:**
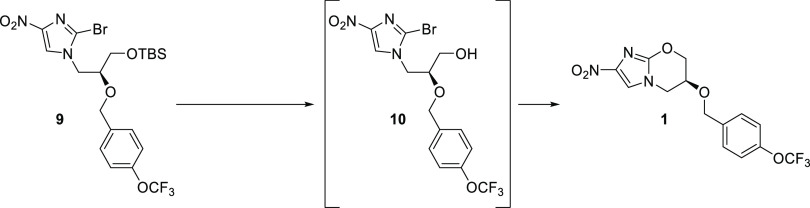
Cyclization to Pretomanid (**4**) Using (*S*)-2-Bromo-1-(3-((*tert*-butyldimethylsilyl)oxy)-2-((4-(trifluoromethoxy)benzyl)oxy)propyl)-4-nitro-1*H*-imidazole (**9**)

entry	solvent	additive	*T* [°C]	*t* [h]	conv. [%] to **1**^a^	result
1	dioxan	HCl + K_2_CO_3_	0	2 h	0	complete conv. to **10**
2	dioxan	HCl + NaH	0 to rt	4 h	0	complete conv. to **10**
3	THF	KF + TBACl	40	12 h	2	complete conv. to **10**
4	DMF	KF + TBACl	40	12 h	3	complete conv. to **10**
5	THF	TBAF (THF)	–5	2 h	0	complete conv. to **10**
6	DMF	TBAF (THF)	–5	2 h	0	complete conv. to **10**
7	THF	TBAF (THF)	–5 to 60	5 days	100	
8	DMF	TBAF (THF)	–5 to 60	3 days	100	side product formation

a Determined by HPLC (area % at 315 nm).

When using potassium fluoride to deprotect and cyclize in a one-pot fashion, DMF and THF were tested as solvents, but the conversion to pretomanid (**1**) was slow. By addition of TBACl, complete deprotection could be observed after 12 h at 40 °C. Heating the reaction mixture for cyclization led to slow product formation but side products formed as well due to the long reaction times required. Using a TBAF solution (1 M in THF) at −5 °C, complete deprotection could be observed after 2 h. Direct cyclization after deprotection could be achieved by heating the reaction mixture to reflux for 3–5 days (Table 5 entry 7). In the case of DMF (Table 5 entry 8), side products were formed. When THF was used as a solvent, side product formation was only observed in traces. After workup, the desired product still contained tetrabutylammonium salts and *t*-butyldimethylsilanol as impurities. After the recrystallization from 2-propanol/heptane and washing with water, tetrabutylammonium salts were only present in traces but unfortunately, *t*-butyldimethylsilanol could not be removed without column chromatography. Pretomanid (**1**) was obtained in 31% overall yield.

## 3 Conclusions

**Scheme 6:**
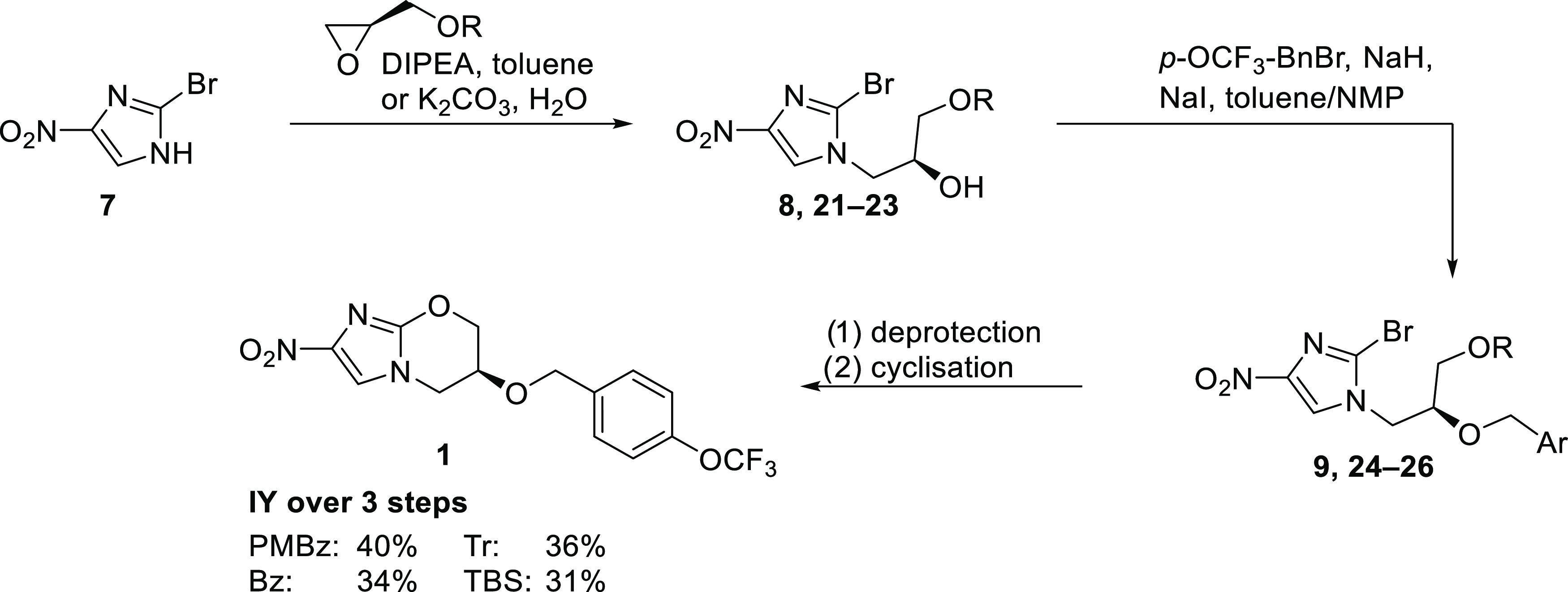
Optimized Synthesis of Pretomanid (**1**)

In variation of Fairlamb’s approach, the synthesis of pretomanid (**1**) from (*R*)-glycidol and 2-bromo-4-nitro-1*H*-imidazole (**7**) using different *O*-protecting groups was investigated with the aim of avoiding purification of intermediates and reducing product loss. Despite the carryover of impurities through each step, pretomanid (**1**) could be isolated in 30–40% yield over three steps in a purity over 99% (Scheme 6).

In this direct comparison, the PMBz route proved to be the most practical route so far with an overall yield of 40% and a purity of 99.7% (HPLC, 315 nm).

Most of the overall yield is lost in the alkylation and the one-pot deprotection/cyclization step. Improvement of these transformations is challenging due to the readily formed *N-* and *O*-regioisomers and the entropically favored competing cyclization involving the secondary hydroxy group to form the undesired five-membered ring.

## 4 Experimental Section

Chemicals were obtained from commercial suppliers and were used without further purification. Deuterated solvents were purchased from Deutero GmbH (Kastellaun, Germany). Dry solvents were purchased from Acros Organics. Column chromatography was performed using cyclohexane and ethyl acetate which were purchased in technical grade and distilled prior to use. All air- or moisture-sensitive reactions were performed under an inert atmosphere (nitrogen or argon) in oven-dried glassware using Schlenk techniques. Reaction temperatures refer to the temperature of the particular cooling or heating bath. Thin-layer chromatography (TLC) was performed on silica plates (TLC Silica 60 F_254_, Merck). UV active compounds were visualized using UV light (λ = 254 nm and λ = 365 nm). All NMR spectra were recorded on the following spectrometers: Bruker Avance-III HD (^1^H-NMR: 300 MHz, ^13^C-NMR: 75.5 MHz, ^19^F-NMR: 282 MHz), Bruker Avance-II (^1^H-NMR: 400 MHz, ^13^C-NMR: 100.6 MHz, ^19^F-NMR: 377 MHz). Chemical shifts are referenced to residual solvent signals ([D]chloroform: 7.26 and 77.16 ppm, for ^1^H-NMR and ^13^C-NMR, respectively) and reported in parts per million (ppm) relative to tetramethylsilane (^1^H, ^13^C) and trichlorofluoromethane (^19^F). Infrared spectra were recorded on an FTIR spectrometer (Bruker Tensor 27) equipped with a diamond ATR unit. Electrospray ionization (ESI) mass spectra were recorded on a 1200-series HPLC system or a 1260-series Infinity II HPLC system (Agilent-Technologies) with binary pump and integrated diode array detector coupled to an LC/MSD-Trap-XTC ion trap mass spectrometer (Agilent-Technologies) or an LC/MSD Infinitylab LC/MSD (G6125B LC/MSD) quadrupole mass spectrometer. High-resolution mass spectra were recorded on a 6545 Q-ToF-mass spectrometer. For analytical HPLC, an Agilent 1260 Infinity system equipped with a binary pump, a diode array detector, and an LC/MSD InfinityLab LC/MSD (G6125B LC/MSD) mass spectrometer was used. An Ascentis Express C18 column (2.7 μm, 2.1 mm × 30 mm, 40 °C) or an ACE C18 PFP column (3 μm, 4.6 mm × 150 mm, 40 °C) with gradient elution using acetonitrile/water (+0.1% formic acid) and a flow rate of 1.0 mL/min was used. Melting points were measured at a Krüss-Optronic KSP 1 N digital melting point meter.

### 4.1 Procedures and Analytical Data

#### 4.1.1 2-Bromo-4-nitro-1*H*-imidazole (**7**)

Procedure based on an unpublished technical report from AAP Pharma Technologies India.^27^ 4-Nitroimidazole (1.0 kg, 7.75 mol, 1.0 equiv) was dissolved in water (4 L) and to this solution was added HBr (2.45 L, 48% aqueous HBr solution, 2.4 equiv). With mechanical stirring, the mixture was heated to 70–75 °C (oil bath) and H_2_O_2_ (1.73 L, 30% in water, 2.5 equiv) was added dropwise in three portions (3 × 0.8 equiv, added at a rate of 0.58 L/h, with 1 h of stirring between portions). Following a 10 h reaction period at 70–75 °C, the solution was cooled to room temperature and then 0–5 °C and stirred for 1 h. The resulting solid precipitate was filtered off, washed with water, and dried to obtain 2,5-dibromo-4-nitro-1*H*-imidazole (**7b**, 2.06 kg, 86% yield) as an off-white solid (HPLC purity (220 nm), 97.8%). The spectroscopic data are consistent with literature values.^26^

2,5-Dibromo-4-nitro-1*H*-imidazole (**7b**, 1.85 kg, 6.3 mol, 1.0 equiv) was dissolved in DMF (3.7 L) under an N_2_ atmosphere. Sodium iodide (2.149 kg, 13.2 mol, 2.1 equiv) was added, and the suspension was stirred with heating to 120 °C (oil bath). After the solids dissolved, trifluoroacetic acid (1.045 L, 12.6 mol, 2.0 equiv) was added (rate of 0.26 L/h). During the addition of TFA, I_2_ fumes were emitted. The mixture was stirred for an additional 2.5 h at 120 °C, after which it is slowly cooled to room temperature (120–25 °C over 3 h). The reaction mixture was then treated with Na_2_SO_3_ solution (1.29 kg in 9.3 L water, 1.5 equiv), cooled to 0–5 °C, and stirred for 1 h. The resulting solid precipitate was filtered off, washed with water, and dried to obtain 0.951 kg of crude 2-bromo-4-nitro-1*H*-imidazole (**7**) as a light-brown solid. This material was suspended in MeOH (5.7 L) and the solution was stirred at 60–65 °C (oil bath) for 2 h. To the clear solution, water (11.4 L) was added at a rate of 11.4 L/h and the mixture was then slowly cooled to room temperature (65–25 °C over 3 h). With further cooling to 0–5 °C and stirred for 1.5 h, the solids were filtered off, washed with cold water (500 mL), and dried to give 2-bromo-4-nitro-1*H*-imidazole (**7**) as a light-brown solid (0.865 kg, 4.16 mol, 66%; HPLC purity (220 nm), 99.3%). The spectrometric data are consistent with literature values.^26^

#### 4.1.2 (*S*)-*tert*-Butyldimethyl(oxirane-2-ylmethoxy)silane (**19**)

According to a procedure from Miwa et al.,^21^ TBSCl (6.10 g, 40.5 mmol, 1.5 equiv) was dissolved in dry DCM (100 mL) and cooled to 0 °C. Imidazole (2.76 g, 40.5 mmol, 1.5 equiv) and DMAP (164.9 mg, 1.35 mmol, 0.05 equiv) were added and stirred for 5 min. After that, (*R*)-glycidol (98%, 98% ee, 2.00 g, 26.5 mmol, 1.0 equiv) was added slowly and the reaction mixture was stirred for 6 h. The reaction mixture was quenched with sat. ammonium chloride solution, extracted with DCM (3 × 20 mL), and the combined organic phases were dried over magnesium sulfate. The solvent was removed in vacuo and the desired product **19** was collected at 57 °C (12 mbar) as a colorless oil (4.88 g, 25.9 mmol, 98%). *R*_f_ (SiO_2_): 0.47 (*^c^*Hex/EtOAc 10:1). IR (ATR): *ν̅* [cm^–1^] = 3673, 2958, 2929, 2901, 2859, 1472, 1253, 1161, 1136, 1093, 833, 776. ^1^H-NMR, COSY (300 MHz, CDCl_3_): δ/ppm = 3.85 (dd, *J* = 11.9 Hz, 3.2 Hz, 1H, TBSO-CH_2a_), 3.65 (dd, *J* = 11.9 Hz, 4.8 Hz, 1H, TBSO-CH_2b_), 3.08 (dddd, *J* = 4.8 Hz, 4.0 Hz, 3.2 Hz, 2.7 Hz, 1H, CH), 2.76 (dd, *J* = 5.2 Hz, 4.0 Hz, 1H, C CH_2a_), 2.63 (dd, *J* = 5.2, Hz 2.7 Hz, 1H, CH_2b_), 0.90 (s, 9H, ^*t*^butyl), 0.07 (d, *J* = 2.7 Hz, 6H, CH_3_). ^13^C-NMR, HSQC, HMBC (75 MHz, CDCl_3_): δ/ppm = 63.7 (TBSO-CH_2_), 52.4 (CH), 44.5 (CH_2_), 25.9 (CH_3_, ^*t*^butyl), 18.4 (CH, ^*t*^butyl), −5.3 (CH_3a_), −5.4 (CH_3b_). MS (ESI): *m*/*z* (%) = 189.1 (39) [M + H]^+^. [α]_589_^19^ = −0.8° (*c* = 10 mg/mL, CHCl_3_). The analytical data are consistent with literature values.^31^

#### 4.1.3 (*S*)-1-(2-Bromo-4-nitro-1*H*-imidazol-1-yl)-3-((*tert*-butyldimethylsilyl)oxy)propan-2-ol (**8**)

A suspension of 2-bromo-4-nitro-1*H*-imidazole (**7**) (95%, 1.00 g, 4.95 mmol, 1.0 equiv), DIPEA (68 mg, 0.50 mmol, 0.10 equiv), and toluene (20 mL) and water (20 mL) at 60 °C was stirred for 5 min before (*S*)-*tert*-butyldimethyl(oxirane-2-ylmethoxy)silane **19** (1.37 g, 7.29 mmol, 1.5 equiv) was added dropwise. After complete addition, the temperature was raised to 70 °C and the suspension was stirred for 70 h. The resulting solution was cooled to room temperature, diluted with ethyl acetate, and the organic phase was washed with diluted HCl (2 mL in 40 mL of H_2_O) and sat. NaHCO_3_ solution. The organic phase was dried over sodium sulfate, and all volatiles were removed in vacuo. The crude product **8** (2.32 g, HPLC purity: 86%, 315 nm) was used for the next step without any further purification.

To purify the crude product, it could also be taken up in MTBE, and to promote precipitation, *n*-heptane was added. After filtration, the colorless solid was dried and the desired product **8** could be obtained as colorless crystals (1.02 g, 2.76 mmol, 73%) (for this purification method, a 1 g (3.79 mmol) scale reaction was used). *R*_f_ (SiO_2_): 0.31 (^*c*^Hex/EtOAc 5:1). Melting range = 108.3–111.8 °C. IR (ATR): *ν̅* [cm^–1^] = 3362, 3180, 2955, 2929, 2886, 2857, 1551, 1513, 1464, 1126, 848, 835, 776. ^1^H-NMR, COSY (300 MHz, CDCl_3_): δ/ppm = 7.97 (s, 1H, H-4), 4.30–4.13 (m, 1H, H-1_a_′), 4.08–3.91 (m, 2H, H-1_b_′ + H-2′), 3.71 (dd, *J* = 10.3 Hz, 4.4 Hz, 1H, H-3_a_′), 3.60 (dd, *J* = 10.3 Hz, 4.6 Hz, 1H, H-3_b_′), 2.73 (d, *J* = 4.9 Hz, 1H, OH), 0.92 (s, 9H, CH_3_, ^*t*^butyl), 0.10 (s, 6H, Si-(CH_3_)_2_). ^13^C-NMR, HSQC, HMBC (75 MHz, CDCl_3_): δ/ppm = 147.1 (C-3), 123.2 (C-5), 120.7 (C-2), 70.2 (C-2′), 64.1 (C-3′), 51.7 (C-1′), 25.9 (C(*C*H_3_)_3_), 18.3 (*C*(CH_3_)_3_), −5.3 (Si-CH_3_), −5.3 (Si-CH_3_). MS (ESI): *m*/*z* (%) = 380.1 (81) [M + H]^+^, 382.1 (100) [M + H]^+^. [α]_589_^19^ = −14.2° (*c* = 10 mg/mL, CHCl_3_). The analytical data are consistent with literature values.^19^

#### 4.1.4 (*S*)-2-Bromo-1-(3-((*tert*-butyldimethylsilyl)oxy)-2-((4-(trifluoromethoxy)benzyl)oxy)-propyl)-4-nitro-1*H*-imidazole (**9**)

To a cooled mixture of dry toluene (8 mL) and dry NMP (8 mL), NaH (60% in mineral oil, 163 mg, 6.77 mmol, 1.30 equiv) and NaI (701 mg, 4.25 mmol, 1.1 equiv) were added. The suspension was stirred for 2 min, and 4-(trifluoromethoxy)benzyl bromide (1.46 g, 5.73 mmol, 1.1 equiv) was added dropwise. The mixture was stirred for 20 min before a solution of crude (*S*)-1-(2-bromo-4-nitro-1*H*-imidazol-1-yl)-3-((*tert*-butyldimethylsilyl)oxy)propan-2-ol (**8**) (86% (315 nm), 2.03 g) in dry NMP (7 mL) was added via syringe pump (5 mL/h) over 100 min while cooling to 0 °C. After complete addition, the suspension was stirred for 1 h at rt. Acetic acid (1.2 mL, 20.8 mmol, 4.0 equiv) was added, followed by water (20 mL). The organic phase was separated, the aqueous phase was extracted with toluene (3 × 10 mL), and the combined organic phases were dried over Na_2_SO_4_. All volatiles were removed in vacuo. The crude product **9** was obtained as an orange-red sticky oil (2.4 g, HPLC purity: 90%, 315 nm), which was used for the next step without further purification. MS (ESI): *m*/*z* (%) = 554.1 (91) [M + H]^+^, 556.1 (100) [M + H]^+^.

#### 4.1.5 (*S*)-Oxirane-2-yl-methyl 4-Methoxybenzoate (**16**)

In an oven-dried Schenk flask, (*R*)-glycidol (98%, 98% ee, 5.00 g, 66.1 mmol, 1.00 equiv) and NEt_3_ (18.3 mL, 132 mmol, 2.0 equiv) were dissolved in DCM (100 mL) under nitrogen atmosphere and cooled in a NaCl ice bath to −15 °C. *p*-Anisoyl chloride (99%, 9.1 mL, 67 mmol, 1.02 equiv) was added dropwise over 10 min. Cooling was removed, and the mixture was stirred for 2 h at room temperature. The reaction was quenched by adding water (50 mL) while cooling and transferred into a separating funnel. The organic phase was washed with saturated NaHCO_3_ solution (50 mL), dried over Na_2_SO_4_, and filtered. All volatiles were removed in vacuo at 40 °C, and the residue was distilled under high vacuum. The desired product (**16**) was collected as a colorless liquid (11.3 g, 54.3 mmol, 82%), which solidified while storing in a refrigerator. *T*_b_: 144–145 °C (2.2 mbar). *R*_f_ (SiO_2_): = 0.63 (EtOAc/CH = 1:1). IR (ATR): *ν̅* [cm^–1^] = 2969, 1709, 1604, 1510, 1249, 1166, 1099, 1025. ^1^H-NMR, COSY (300 MHz, CDCl_3_): δ/ppm = 8.13–7.99 (m, 2H, H-2,6), 6.94–6.89 (m, 2H, H-3,5), 4.62 (dd, *J* = 12.3 Hz, *J* = 3.1 Hz, 1H, CH_2a_OC=O), 4.13 (dd, *J* = 12.3 Hz, *J* = 6.2 Hz, 1H, CH_2b_OC=O), 3.85 (s, 3H, −OCH_3_), 3.35–3.30 (m, 1H, −CH−), 2.88 (dd, *J* = 4.9 Hz, ^3^*J* = 4.1 Hz, 1H, CH_2a_, oxirane), 2.71 (dd, *J* = 4.9 Hz, *J* = 2.6 Hz, 1H, CH_2b_, oxirane). ^13^C-NMR, HMBC, HSQC (75 MHz, CDCl_3_): δ = 166.1 (C=O), 163.7 (C-4), 131.9 (C-2,6), 122.1 (C-1), 113.8 (C-3,5), 65.3 (**C**H_2_OC=O), 49.7 (−CH−), 44.8 (CH_2_, oxirane). ESI-HRMS: calcd for [M + Na]^+^: *m*/*z* = 231.0628, found: *m*/*z* = 231.0627. [α]_589_^19^ = +30.0 (CHCl_3_, *c* = 10 mg/mL).

#### 4.1.6 (*S*)-3-(2-Bromo-4-nitro-1*H*-imidazol-1-yl)-2-hydroxypropyl 4-Methoxybenzoate (**21**)

A round-bottom flask was charged with imidazole (**7**) (95%, 1.00 g, 4.95 mmol, 1.00 eq.), K_2_CO_3_ (0.068 g, 0.495 mmol, 0.10 equiv), and water (20 mL). The suspension was heated to 55 °C and stirred for 5 min before (*S*)-oxirane-2-yl-methyl 4-methoxybenzoate (**16**) (1.44 g, 6.92 mmol, 1.40 equiv) was added in one portion. The slight greenish suspension was stirred for 44 h at 55 °C. After cooling to room temperature, the mixture was vacuum-filtered and carefully washed with water (4 × 4 mL). The slight yellowish solid was dried in the air overnight and then in a desiccator for 2 days. The crude product **21** (1.99 g, HPLC purity: 87%, 315 nm) was used for the next step without any further purification. Characteristic NMR signals: ^1^H-NMR (300 MHz, CDCl_3_): δ/ppm = 8.01 (s, 1H), 8.00–7.95 (m, 2H), 7.0–6.92 (m, 2H), 4.54–4.25 (m, 4H), 4.19–4.04 (m, 1H), 3.88 (s, 3H), 3.29 (s, 1H). ^13^C-NMR (75 MHz, CDCl_3_): δ/ppm = 166.6, 164.1, 147.1, 132.0, 123.3, 121.3, 120.6, 114.1, 68.6, 65.7, 55.7, 51.8. MS (ESI): *m*/*z* (%) = 399.9 (100) [M + H]^+^, 402. (100) [M + H]^+^.

#### 4.1.7 (*S*)-3-(2-Bromo-4-nitro-1*H*-imidazol-1-yl)-2-((4-(trifluoromethoxy)benzyl)oxy)propyl 4-Methoxybenzoate (**24**)

An oven-dried Schlenk flask was charged, while cooling in an ice bath, with NaH (60% in mineral oil, 0.23 g, 5.63 mmol, 1.30 equiv), dry toluene (7 mL), NaI (99%, 0.68 g, 4.54 mmol, 1.05 equiv), and dry NMP (7 mL) under nitrogen atmosphere. 4-(Trifluoromethoxy)benzyl bromide (98%, 0.73 mL, 4.54 mmol, 1.05 equiv) was added dropwise over 10 min to the suspension while cooling. The mixture was stirred for further 10 min before a solution of crude **21** (83% (315 nm), 1.99 g) in dry NMP (7 mL) was added via a syringe pump (5 mL/h) over 100 min while keeping the temperature between 1 and 3 °C. After complete addition, the suspension was stirred for further 10 min (LC-MS showed complete conversion). Acetic acid (0.99 mL, 17.3 mmol, 4.0 equiv) was added, followed by water (14 mL). The mixture was transferred into a separating funnel, and the organic phase was separated. The aqueous phase was extracted with toluene (2 × 10 mL), and the combined organic phases were dried over Na_2_SO_4_. After filtration, all volatiles were removed first at 40–50 °C (16 mbar) and then at 80–90 °C (0.5 mbar). The crude product **24** was obtained as an orange-red sticky solid (2.57 g, HPLC purity: 82%, 315 nm), which was used for the next step without further purification. Characteristic NMR signals: ^1^H-NMR (300 MHz, CDCl_3_): δ/ppm = 8.03–7.92 (m, 2H), 7.86 (s, 1H), 7.24–7.18 (m, 2H), 7.17–7.12 (m, 3H), 7.00–6.89 (m, 3H), 4.71 (d, *J* = 11.7 Hz, 1H), 4.50–4.40 (m, 5H), 4.29 (dd, *J* = 14.6 Hz, 3.0 Hz, 1H), 4.15 (dd, *J* = 14.6 Hz, 8.5 Hz, 1H), 4.05–3.92 (m, 2H), 3.88 (s, 3H). ^13^C-NMR (75 MHz, CDCl_3_): δ/ppm = 165.8, 164.1, 149.3, 147.4, 135.2, 131.9, 129.6, 122.9, 121.4, 121.3, 120.51 (q, *J* = 257.6 Hz, the outer signals of the CF_3_ resonance are not visible), 120.4, 114.1, 74.9, 71.8, 62.1, 55.7, 50.8. ^19^F-NMR (282 MHz, CDCl_3_): δ/ppm = −57.9 (CF_3_). MS (ESI): *m*/*z* (%) = 574.1 (100) [M + H]^+^, 576.1 (98) [M + H]^+^.

#### 4.1.8 (*S*)-Oxirane-2-yl-methyl Benzoate (**17**)

According to a procedure from Scolastico et al.,^32^ in an oven-dried Schenk flask, (*R*)-glycidol (98%, 98% ee, 3.00 g, 39.7 mmol, 1.0 equiv) and NEt_3_ (11.2 mL, 80.8 mmol, 2.0 equiv) were dissolved in DCM (60 mL) under nitrogen atmosphere and cooled in a NaCl ice bath to −13 °C. Benzoyl chloride (99%, 4.7 mL, 40 mmol, 1.02 equiv) was added dropwise over 10 min. Cooling was removed and the mixture was stirred for 3 h at room temperature. The reaction was quenched by adding water (50 mL) while cooling and transferred into a separating funnel. The organic phase was washed with saturated NaHCO_3_ solution (50 mL), dried over Na_2_SO_4_, and filtered. All volatiles were removed in vacuo at 40 °C, and the residue was distilled under high vacuum. The desired product **17** was collected as a colorless liquid (5.95 g, 33.4 mmol, 84%). *T*_b_: 73–75 °C (0.2 mbar). Lit.: 170 °C (1.1 mbar).^32^*R*_f_ (SiO_2_): 0.60 (EtOAc/cyclohexane = 1:2). IR (ATR): *ν̅* [cm^–1^] = 3063, 1720, 1452, 1272, 1111, 1071. ^1^H-NMR, COSY (300 MHz, CDCl_3_): δ/ppm = 8.10–8.03 (m, 2H, H-2,6), 7.62–7.54 (m, 1H, H-4), 7.49–7.41 (m, 2H, H-3,5), 4.66 (dd, *J* = 12.3 Hz, *J* = 3.1 Hz, 1H, CH_2a_OC=O), 4.18 (dd, *J* = 12.3 Hz, *J* = 6.2 Hz, 1H, CH_2b_OC=O), 3.39–3.31 (m, 1H, −CH−), 2.90 (dd, *J* = 4.9 Hz, *J* = 4.1 Hz, 1H, H_a_-CH_2_, oxirane), 2.74 (dd, *J* = 4.9 Hz, *J* = 2.6 Hz, 1H, H_b_-CH_2_, oxirane). ^13^C-NMR, HMBC, HSQC (75 MHz, CDCl_3_): δ/ppm = 166.4 (C=O), 133.4 (C-4), 129.9 (C-2,6), 129.8 (C-1), 128.6 (C-3,5), 65.6 (**C**H_2_OC=O), 49.6 (−CH−), 44.9 (CH_2_, oxirane). MS (ESI): *m*/*z* (%) = 179.1 (100%, [M + H]^+^). [α]_589_^19^ = 2.9 (CHCl_3_, *c* = 10 mg/mL). The analytical data are consistent with literature values.^32^

#### 4.1.9 (*S*)-3-(2-Bromo-4-nitro-1*H*-imidazol-1-yl)-2-hydroxypropyl Benzoate (**22**)

A round-bottom flask was charged with imidazole **7** (95%, 1.00 g, 4.95 mmol, 1.00 equiv), DIPEA (0.26 mL, 1.59 mmol, 0.30 equiv), and dry toluene (13 mL) under nitrogen atmosphere. The suspension was heated to 70 °C and stirred for 5 min before oxirane **17** (2.19 g, 6.92 mmol, 1.40 equiv), dissolved in dry toluene (5 mL), was added via a syringe pump (0.3 mL/min) over 20 min. The slight greenish suspension was stirred for 46 h at 70 °C. After cooling to room temperature, the solvent was removed in vacuo and the residue was redissolved in warm EtOAc (20 mL). The solution was washed once with sat. NH_4_Cl solution and once with sat NaHCO_3_ solution (15 mL each). After drying over Na_2_SO_4_ and filtration, all volatiles were removed in vacuo at 40 °C. The obtained crude product **22** (1.58 g, HPLC purity: 87%, 315 nm) was used for the next step without any further purification. Characteristic NMR signals: ^1^H-NMR (300 MHz, CDCl_3_): δ/ppm = 8.10–7.96 (m, 3H), 7.71–7.58 (m, 1H), 7.54–7.43 (m, 2H), 4.55–4.47 (m, 1H), 4.43 (d, *J* = 5.2 Hz, 1H), 4.40–4.32 (m, 2H), 4.31–4.27 (m, 1H), 4.12 (dd, *J* = 14.3 Hz, 7.8 Hz, 1H), 3.16 (s, 1H). ^13^C-NMR (75 MHz, CDCl_3_): δ/ppm = 166.8, 147.3, 134.0, 129.9, 129.0, 128.8, 123.1, 120.6, 68.7, 65.9, 51.6. MS (ESI): *m*/*z* (%) = 370.0 (100) [M + H]^+^, 372.0 (99) [M + H]^+^.

#### 4.1.10 (*S*)-3-(2-Bromo-4-nitro-1*H*-imidazol-1-yl)-2-((4-(trifluoromethoxy)benzyl)oxy)propyl Benzoate (**25**)

An oven-dried Schlenk flask was charged, while cooling in an ice bath, with NaH (60% in mineral oil, 0.23 g, 5.63 mmol, 1.30 equiv), dry toluene (7 mL), NaI (99%, 0.68 g, 4.54 mmol, 1.05 equiv), and dry NMP (7 mL) under nitrogen atmosphere. 4-(Trifluoromethoxy)benzyl bromide (98%, 0.73 mL, 4.54 mmol, 1.05 equiv) was added dropwise over 10 min to the suspension while cooling. The mixture was stirred for further 10 min before a solution of crude **22** (1.58 g), 87% (315 nm) in dry NMP (7 mL) was added via a syringe pump (5 mL/h) over 100 min while keeping the temperature between 1 and 3 °C. After complete addition, the suspension was stirred for further 10 min (LC-MS showed complete conversion). Acetic acid (0.99 mL, 17.3 mmol, 4.0 equiv) was added, followed by water (14 mL). The mixture was transferred into a separating funnel, and the organic phase was separated. The aqueous phase was extracted with toluene (2 × 10 mL), and the combined organic phases were dried over Na_2_SO_4_. After filtration, all volatiles were removed first at 40–50 °C (16 mbar) and then at 80–90 °C (0.5 mbar). The crude product **25** was obtained as an orange-red sticky solid (1.91 g, 84%, 315 nm), which was used for the next step without further purification. Characteristic NMR signals: ^1^H-NMR (300 MHz, CDCl_3_): δ/ppm = 8.09–7.99 (m, 2H), 7.86 (s, 1H), 7.68–7.58 (m, 1H), 7.54–7.43 (m, 2H), 7.23–7.08 (m, 4H), 4.72 (d, *J* = 11.7 Hz, 1H), 4.56–4.43 (m, 3H), 4.32 (dd, *J* = 14.5 Hz, 3.1 Hz, 1H), 4.16 (dd, *J* = 14.5 Hz, 8.6 Hz, 1H), 4.05–3.95 (m, 1H). ^13^C-NMR (75 MHz, CDCl_3_): δ/ppm = 166.1, 149.4, 149.3, 147.4, 135.1, 133.9, 129.8, 129.6, 129.2, 128.8, 122.9, 121.3, 120.50 (q, *J* = 257.8 Hz, the outer signals of the CF_3_ resonance are not visible), 120.4, 74.8, 71.8, 62.4, 50.7. ^19^F-NMR (282 MHz, CDCl_3_): δ/ppm = −57.9 (CF_3_). MS (ESI): *m*/*z* (%) = 544.1 (99) [M + H]^+^, 546.0 (100) [M + H]^+^.

#### 4.1.11 (*S*)-2-((Trityloxy)methyl)oxirane (**18**)

According to a procedure from Schweizer et al.,^30^ in an oven-dried Schenk flask, trityl chloride (16.30 g, 58.5 mmol, 1.1 equiv) was dissolved in dry DCM (60 mL) under nitrogen atmosphere. While cooling in an ice bath, a solution of (*R*)-glycidol (98%, 98% ee, 4.00 g, 52.9 mmol, 1.0 equiv) and NEt_3_ (16:3 mL, 117 mmol, 2.0 equiv) in dry DCM (30 mL) was added. After the addition of DMAP (0.01 equiv), the cooling bath was removed and the solution was stirred for 24 h at room temperature. The reaction was quenched by the addition of sat. NH_4_Cl-solution (400 mL), the organic phase was separated and the aqueous phase was extracted with Et_2_O (3 × 250 mL). The combined organic phases were washed with brine (3 × 500 mL) and dried over Na_2_SO_4_. The crude product was used for the next step without further purification. Melting range: 100.1–101.4 °C, Lit.: 94–96 °C.^30^*R*_f_ (SiO_2_): 0.63 (EtOAc/cyclohexane = 1:5). IR (ATR): *ν̅* [cm^–1^] = 3058, 3031, 2923, 2871, 1491, 1448, 1070, 1033. ^1^H-NMR, COSY (300 MHz, CDCl_3_): δ/ppm = 7.50–7.45 (m, H-2,6, 6H, trityl), 7.34–7.28 (m, H-3,5, 6H, trityl), 7.34–7.28 (m, H-4, 3H, trityl), 3.34 (dd, *J* = 10.0 Hz, *J* = 2.3 Hz, CH_2a_-OTr, 1H), 3.19–3.15 (m, −CH–, 1H), 3.13 (dd, *J* = 10.0 Hz, *J* = 5.4 Hz, CH_2b_-OTr, 1H), 2.78 (dd, *J* = 5.1 Hz, *J* = 4.1 Hz, H_a_-3, 1H), 2.63 (dd, *J* = 5.1 Hz, *J* = 2.5 Hz, H_b_-3, 1H). ^13^C-NMR, HMBC, HSQC (75 MHz, CDCl_3_): δ/ppm = 143.9 (C-1, trityl), 128.8 (C-2,6, trityl), 128.0 (C-3,5, trityl), 127.2 (C-4, trityl), 86.8 (−**C**(Ph)_3_), 64.9 (**C**H_2_-OTr), 51.2 (−CH−), 44.8 (C-3). MS (ESI): *m*/*z* (%) = 339.0 (100%, [M + Na]^+^). [α]_589_^19^ = +8.7 (CHCl_3_, *c* = 10 mg/mL). The analytical data are consistent with literature values.^30^

#### 4.1.12 (*S*)-1-(2-Bromo-4-nitro-1*H*-imidazol-1-yl)-3-(trityloxy)propan-2-ol (**23**)

A round-bottom flask was charged with imidazole **7** (95%, 1.00 g, 4.95 mmol, 1.00 equiv), DIPEA (0.26 mL, 1.59 mmol, 0.30 equiv), and dry toluene (13 mL) under nitrogen atmosphere. The suspension was heated to 70 °C and stirred for 5 min before oxirane **18** (2.19 g, 6.92 mmol, 1.40 equiv), dissolved in dry toluene (5 mL), was added via a syringe pump (0.3 mL/min) over 20 min. The slight greenish suspension was stirred for 46 h at 70 °C. After cooling to room temperature, the solvent was removed in vacuo and the residue was redissolved in warm EtOAc (20 mL). The solution was washed once with sat. NH_4_Cl solution and once with sat NaHCO_3_ solution (15 mL each). After drying over Na_2_SO_4_ and filtration, all volatiles were removed in vacuo at 40 °C. The obtained crude sticky oil **23** (2.90 g, HPLC purity: 82%, 315 nm) was used for the next step without any further purification. Characteristic NMR signals: ^1^H-NMR (300 MHz, CDCl_3_): δ/ppm = 7.84 (s, 1H), 7.46–7.38 (m, 6H), 7.38–7.24 (m, 9H), 4.27–4.15 (m, 1H), 4.08–3.98 (m, 1H), 3.30 (dd, *J* = 9.9 Hz, 4.8 Hz, 1H), 3.24–3.11 (m, 1H), 2.49 (d, *J* = 4.5 Hz, 1H). ^13^C-NMR (75 MHz, CDCl_3_): δ/ppm = 143.2, 128.6, 128.3, 127.7, 123.0, 120.5, 87.6, 69.4, 64.5, 51.6. MS (ESI): *m*/*z* (%) = 508.1 (99) [M + H]^+^, 510.1 (100) [M + H]^+^.

#### 4.1.13 (*S*)-2-Bromo-4-nitro-1-(2-((4-(trifluoromethoxy)benzyl)oxy)-3-(trityloxy)propyl)-1*H*-imidazole (**26**)

An oven-dried Schlenk flask was charged, while cooling in an ice bath, with NaH (60% in mineral oil, 0.23 g, 5.63 mmol, 1.30 equiv), dry toluene (7 mL), NaI (99%, 0.68 g, 4.54 mmol, 1.05 equiv), and dry NMP (7 mL) under nitrogen atmosphere. 4-(Trifluoromethoxy)benzyl bromide (98%, 0.73 mL, 4.54 mmol, 1.05 equiv) was added dropwise over 10 min to the suspension while cooling. The mixture was stirred for further 10 min before a solution of crude **23** (2.90 g), 82% (315 nm) in dry NMP (7 mL) was added via a syringe pump (5 mL/h) over 100 min while keeping the temperature between 1 and 3 °C. After complete addition, the suspension was stirred for further 10 min (LC-MS showed complete conversion). Acetic acid (0.99 mL, 17.3 mmol, 4.0 equiv) was added, followed by water (14 mL). The organic phase was separated, and the aqueous phase was extracted with toluene (2 × 10 mL). The combined organic phases were dried over Na_2_SO_4_, and all volatiles were removed first at 40–50 °C (16 mbar) and then at 80–90 °C (0.5 mbar). The crude product **26** was obtained as an orange-red sticky solid (3.52 g, 73%, 315 nm), which was used for the next step without further purification. Characteristic NMR signals: ^1^H-NMR (300 MHz, CDCl_3_): δ/ppm = 7.75 (s, 1H), 7.57–7.40 (m, 6H), 7.38–7.22 (m, 11H), 7.21–7.06 (m, 4H), 4.52 (d, *J* = 11.8 Hz, 1H), 4.38–4.30 (m, 1H), 4.30–4.25 (m, 1H), 4.10 (dd, *J* = 14.4 Hz, 7.9 Hz, 1H), 3.75–3.58 (m, 1H), 3.26 (d, *J* = 5.1 Hz, 2H). ^13^C-NMR (75 MHz, CDCl_3_): δ/ppm = 149.2, 147.2, 143.4, 135.6, 129.4, 128.6, 128.2, 127.2, 122.9, 122.2, 121.2, 120.5, 120.5 (q, *J* = 257.5 Hz, the outer signals of the CF_3_ resonance are not visible), 118.8, 87.6, 76.1, 71.6, 62.0, 50.4. ^19^F-NMR (282 MHz, CDCl_3_): δ/ppm = −57.87 (CF_3_). MS (ESI): *m*/*z* (%) = 682.1 (37) [M + H]^+^, 683.1 (16) [M + H]^+^, 684.1 (41) [M + H]^+^, 685.1 (15) [M + H]^+^.

#### 4.1.14 Optimized Procedure for (*S*)-2-Nitro-6-((4-(trifluoromethoxy)benzyl)oxy)-6,7-dihydro-5*H*-imidazo[2,1-*b*][1,3]oxazine (Pretomanid) (**1**) Using the PMBz Route

Crude **24** (82% (315 nm), 2.57 g) was dissolved in dry methanol (20 mL) under nitrogen atmosphere and cooled to −10 °C using a cryostat. K_2_CO_3_ (7.60 g, 55.0 mmol, 15 equiv) was added in one portion, and the suspension was stirred for 2 h at −10 °C. Then, the cryostat temperature was changed to 0 °C and the suspension was stirred for 22 h (LC-MS showed complete conversion). The reaction was quenched by the addition of water (40 mL) and stirred for 23 h at room temperature. The precipitated orange-brown solid was vacuum-filtered and carefully washed with water (4 × 5 mL). The solid was dried in air overnight, then in a desiccator overnight. After drying, the solid was suspended in MTBE (7 mL), heated to reflux, and cooled to room temperature. The colorless solid was filtered, washed with MTBE (3 × 1 mL), and dried in vacuo at 40 °C (0.72 g, 2.00 mmol, 40% (related to 4.95 mmol imidazole **7**)). *R*_f_ (SiO_2_) = 0.38 (^*c*^Hex/EtOAc 1:8). Melting range = 149.3–150.7 °C. Lit.: 149–150 °C.^21^ IR (ATR): *ν̅* [cm^–1^] = 3132, 2987, 2881, 1579, 1540, 1498, 1470, 1275, 1266, 1157, 827. ^1^H-NMR, COSY (300 MHz, CDCl_3_): δ/ppm = 7.41 (s, 1H, H-3), 7.38–7.30 (m, 2H, *o*-C, Ph-OCF_3_), 7.25–7.14 (m, 2H, *m*-C, Ph-OCF_3_), 4.72 (d, *J* = 11.8 Hz, CH_2a_-Ph-OCF_3_), 4.62 (d, *J* = 11.8 Hz, CH_2b_-Ph-OCF_3_), 4.66–4.58 (m, 1H, H-7_b_), 4.37 (dd, *J* = 12.2 Hz, 1.5 Hz, 1H, H-7_a_), 4.29–4.17 (m, 2H, H-5), 4.17–4.11 (m, 1H, H-6). ^13^C-NMR, HSQC, HMBC (75 MHz, CDCl_3_): δ/ppm = 149.3 (*p*-C, Ph-OCF_3_), 147.2 (C-9), 143.8 (C-2), 135.3 (*ipso*-C, Ph-OCF_3_), 129.3 (*o*-C, Ph-OCF_3_), 120.5 (q, *J* = 258 Hz, OCF_3_, the outer signals of the CF_3_ resonance are not visible), 121.4 (*m*-C, Ph-OCF_3_), 115.3 (C-3,), 70.3 (CH_2_-Ph-OCF_3_), 67.3 (C-7), 66.7 (C-6), 47.7 (C-5). ^19^F-NMR (282 MHz, CDCl_3_): δ/ppm = −57.9 (CF_3_). MS (ESI): *m*/*z* (%) = 360.1 (100) [M + H]^+^. [α]_589_^19^ = −29.2° (*c* = 10 mg/mL, CHCl_3_). The analytical data are consistent with literature values.^21^

## Supporting Information Available

Optimization studies, chromatograms, and NMR spectra (PDF)
