# Supplementary material for: Short and Efficient Synthesis of the Antituberculosis Agent Pretomanid from (R)-Glycidol
Source: Org Process Res Dev. 2023 Sep 5;27(9):1641–51. doi: 10.1021/acs.oprd.3c00187 (PMC10510727; doi:10.1021/acs.oprd.3c00187)
Supplement: Supplementary file 1 — op3c00187_si_001.pdf [file op3c00187_si_001.pdf]

Supporting Information

For

Short and Efficient Synthesis of the Anti-Tuberculosis Agent Pretomanid from (*R*)-Glycidol

Tobias Lucas, [a]+ *Jule-Philipp Dietz,* [a]+ *Flavio S. P. Cardoso,* [b] *David R. Snead,* [b] *Ryan C. Nelson,* [b] *Kai O. Donsbach,* [b] *B. Frank Gupton,* [b] *Till Opatz**[a]

+ *These authors contributed equally to this work.*

[a] Department of Chemistry, Johannes Gutenberg-University, Duesbergweg 10–14, 55128 Mainz, Germany

[b] Department of Chemical and Life Sciences Engineering, Virginia Commonwealth University, Richmond, Virginia 23284, United States

TBS-Route:
(*S*)-2-Nitro-6-((4-(trifluoromethoxy)benzyl)oxy)-6,7-dihydro-5*H*-imidazo[2,1-b][1,3]oxazine (pretomanid) (1)

(*S*)-2-Bromo-1-(3-((*tert*-butyldimethylsilyl)oxy)-2-((4-(trifluoromethoxy)benzyl)oxy)propyl)-4-nitro-1*H*-imidazole (**9**, 90%, 2.4 g) was dissolved in dry THF (40 mL) under nitrogen atmosphere and cooled in a NaCl-ice bath. Tetrabutylammonium fluoride solution (15.6 mL, 1M in THF) was added dropwise to the stirred solution and the mixture was further stirred for 30 min at –10 °C until LC-MS showed complete cleavage of the protecting group. The temperature was raised to 66 °C and the mixture was stirred for 36 h. After full conversion, the mixture was cooled to rt, quenched with water. The resulting brown precipitate was washed with water, dried in vacuo and recrystallized from MTBE/heptane. The crude product was purified by column chromatography (^{c}Hex/EtOAc 1:8) and obtained as slightly yellow solid (541 mg, 1.52 mmol, 31% related to 4.95 mmol imidazole **7**) in 99.1% purity (HPLC, 315 nm).

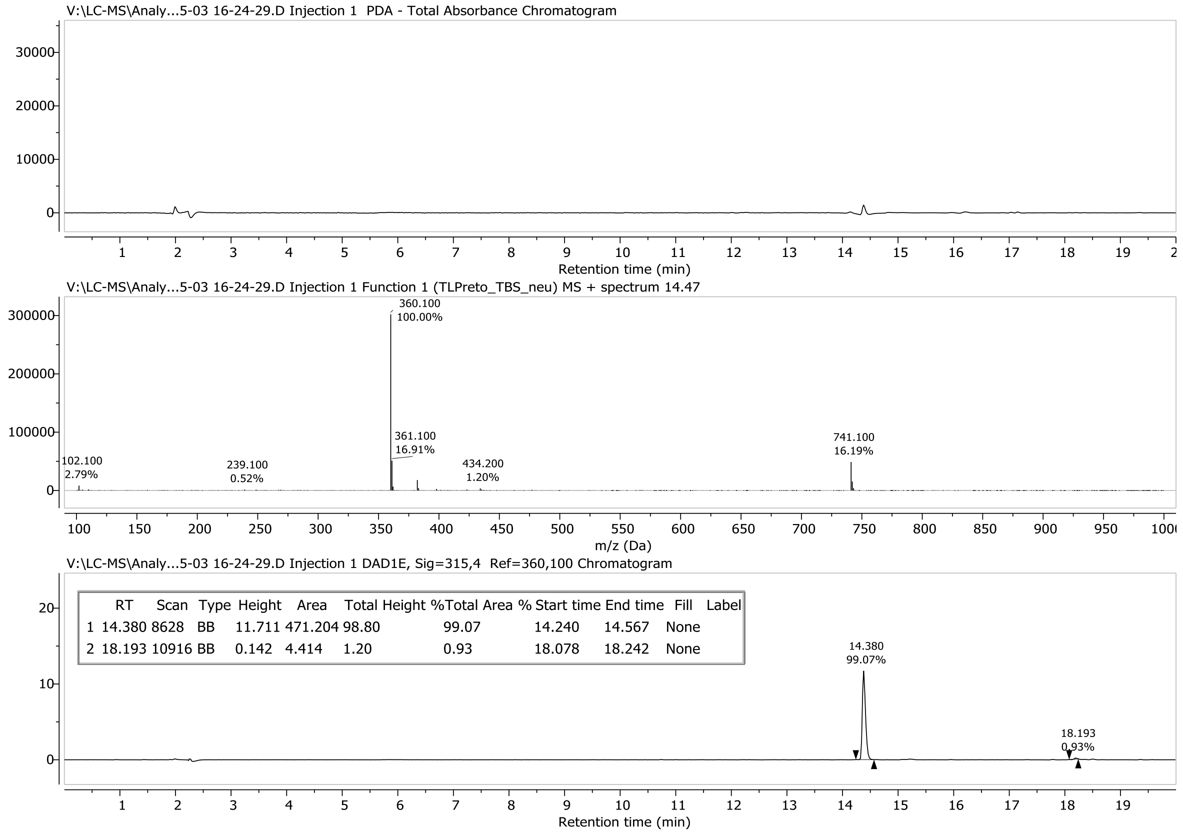

Figure S1. HPLC chromatogram (column: ACE C18 PFP, gradient MeCN:H2O (+ 0.1% formic acid): 5:95 (2.5 min), 5:95 to 95:5 (15 min), 95:5 (2.5 min), λ = 315 nm) of pretomanid **1** prepared through the TBS-route.

PMBz-Route:

(*S*)-2-Nitro-6-((4-(trifluoromethoxy)benzyl)oxy)-6,7-dihydro-5*H*-imidazo[2,1-b][1,3]oxazine (pretomanid) (1)

Screening one pot deprotection and cyclization towards pretomanid:

Table S1. Temperature screening for the saponification and cyclization of **24** yielding pretomanid.

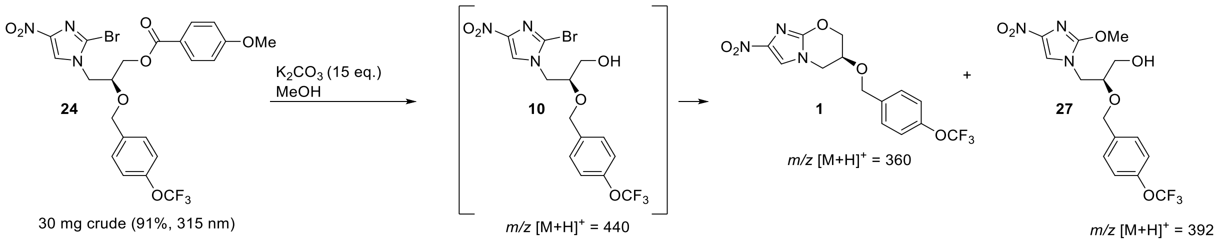

#	*T* [°C]	*t* [h]	**area% (315 nm)**				
			27	10	1	24	BPs
**1**	r.t.	24	57	-	43	-	
**2**	2-6	3	44	2	54	-	
**3**[a]	-10	1	-	88	traces	12	
	-10	2	-	86	14	-	
	-10	3	-	85	15	-	
	-10	24	56	4	32	12	
**4**[a]	-10	2					
	-5	22	11	12	65	-	13
**5**[a]	-10	2					
	0	4	2	36	55	-	8
	0	24	7	2	82	-	9
	0	48	17		71	-	11
	r.t.	72	72	-	20	-	8

[a] cooled with a cryostat, BPs = byproducts

Crude **24** (82% (315 nm), 2.57 g) was dissolved in dry methanol (20 mL) under nitrogen atmosphere and cooled to -10 °C using a cryostat. K_2CO_3 (7.60 g, 55.0 mmol, 15 eq.) was added in one portion and the suspension stirred for two hours at -10 °C. Then, the cryostat temperature was changed to 0 °C and the suspension stirred for 22 h (LC-MS showed complete conversion).

The reaction was quenched by the addition of water (40 mL) and stirred for 23 h at room temperature. The precipitated orange-brown solid was vacuum-filtered and carefully washed with water (4 x 5 mL). The solid was dried on the air overnight, then in a desiccator overnight. After drying, the solid was suspended in MTBE (7 mL), heated to reflux and cooled to room temperature. The colorless solid was filtered, washed with MTBE (3 x 1 mL) and dried in vacuo at 40 °C (0.72 g, 2.00 mmol, 40% (related to 4.95 mmol imidazole **7**)) in 99.7% purity (HPLC, 315 nm).

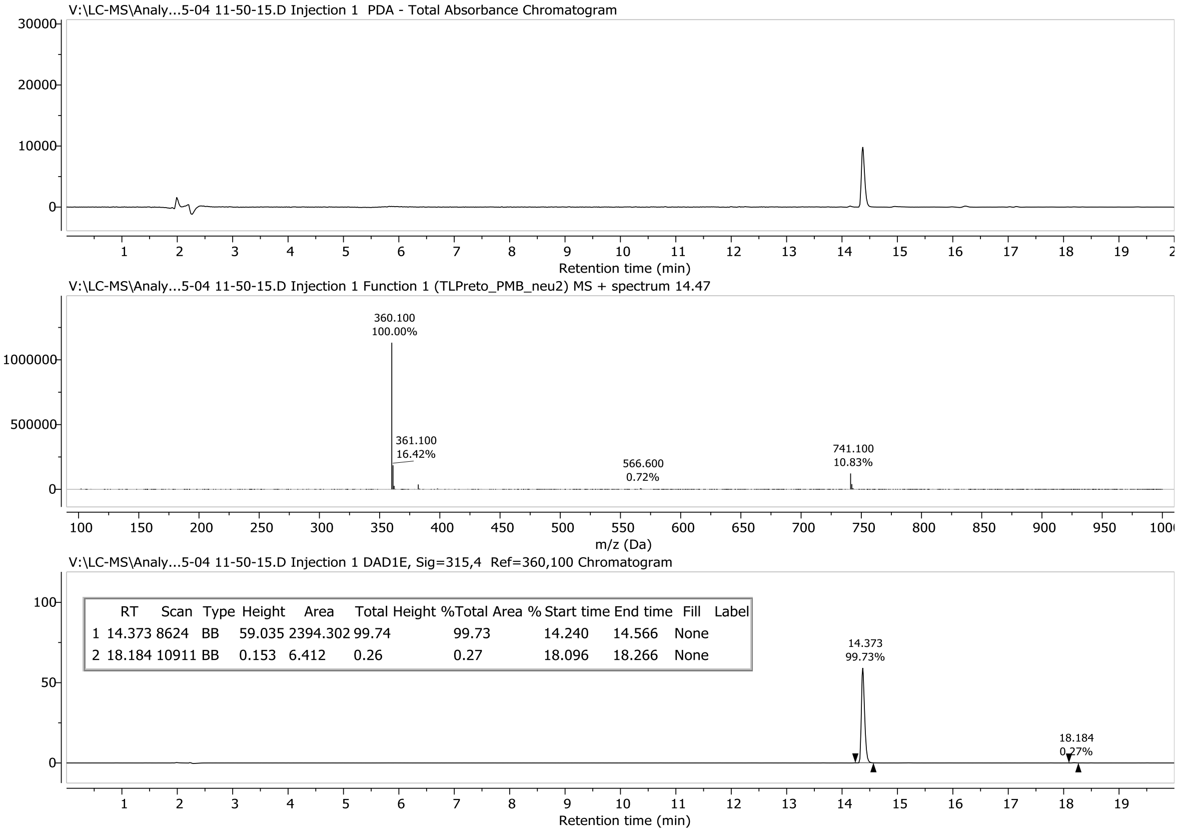

Figure S2. HPLC chromatogram (column: ACE C18 PFP, gradient MeCN:H2O (+ 0.1% formic acid): 5:95 (2.5 min), 5:95 to 95:5 (15 min), 95:5 (2.5 min), λ = 315 nm) of pretomanid **1** prepared through the PMB-route.

Bz-Route:

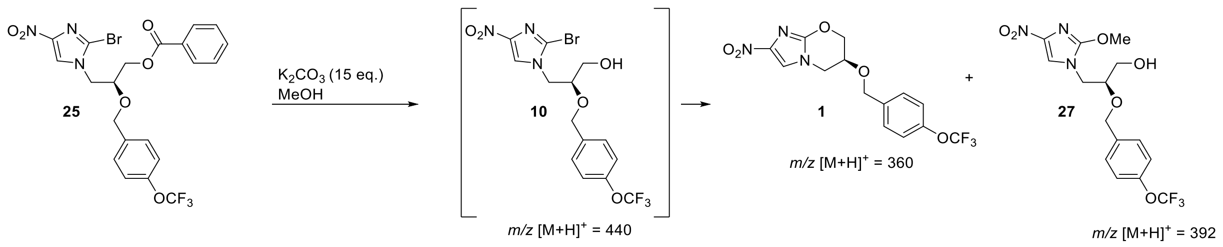

Figure S3: Saponification and cyclization of **25**.

(*S*)-2-Nitro-6-((4-(trifluoromethoxy)benzyl)oxy)-6,7-dihydro-5*H*-imidazo[2,1-b][1,3]oxazine (pretomanid) (1)

Crude **25** (84% (315 nm), 1.91 g) was dissolved in dry methanol (20 mL) under nitrogen atmosphere and cooled to -15 °C using a cryostat. K_2CO_3 (6.11 g, 44.2 mmol, 15 eq.) was added in one portion and the suspension stirred for 15 min at this temperature. Then, cooling was removed and the suspension stirred for 90 min to room temperature. (LC-MS showed complete conversion of starting material). The reaction mixture was quenched by the addition of water (40 mL) and stirred for 17 h at room temperature. The precipitated oily orange-brown solid was vacuum-filtered and carefully washed with water (4 x 5 mL). The solid was air-dried overnight, then in a desiccator overnight. After drying, the solid was suspended in MTBE (7 mL), heated to reflux for 2 min and cooled to r.t. The colorless solid was filtered, washed with MBE (3 x 1 mL) and dried in vacuo at 40 °C (0.78 g, 1.34 mmol, 34% (related to 4.95 mmol **7**)) in 99.4% purity (HPLC, 315 nm).

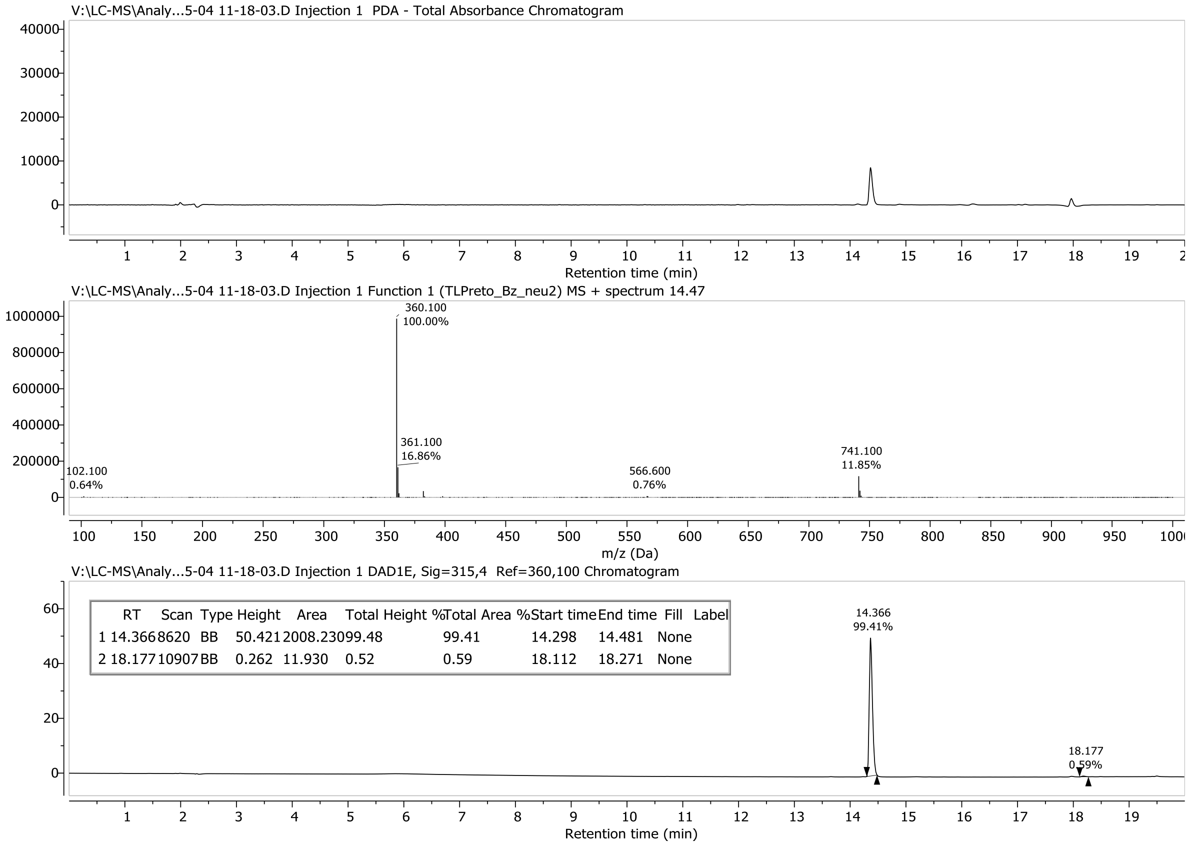

Figure S4. HPLC chromatogram (column: ACE C18 PFP, gradient MeCN:H2O (+ 0.1% formic acid): 5:95 (2.5 min), 5:95 to 95:5 (15 min), 95:5 (2.5 min), λ = 315 nm) of pretomanid **1** prepared through the Bz-route.

Trityl-Route:

(*S*)-2-Nitro-6-((4-(trifluoromethoxy)benzyl)oxy)-6,7-dihydro-5*H*-imidazo[2,1-b][1,3]oxazine (pretomanid) (1)

Previous investigation:

In contrast to the previously described ester protecting groups, the trityl group has to be removed under acidic conditions. Thus, to a solution of crude **26** in MeOH, methanolic HCl (3N) was added at r.t.. After one hour, 96% conversion to the free alcohol **10** could be detected by LC-MS. Using

the standard conditions for the cyclisation (adding K_2CO_3) also led to the same byproduct formation as in the other routes. Using a temperature gradient as developed for the PMBz route led to more promising results (Table 2). It was conspicuous that the reaction rate was slower than the one using PMBz. After 67 h at 0 °C, 63% pretomanid (**1**) could be detected along with 4% of remaining alcohol **10** and 6% of undesired compound **27** Further stirring for 24 h led to complete conversion of **10** but also to more pronounced byproduct formation and an overall diminished conversion to pretomanid (**1**) (57%).

Table S2. Synthesis of pretomanid through trityl-precursor **26** using HCl first, then K_2CO_3 in MeOH.

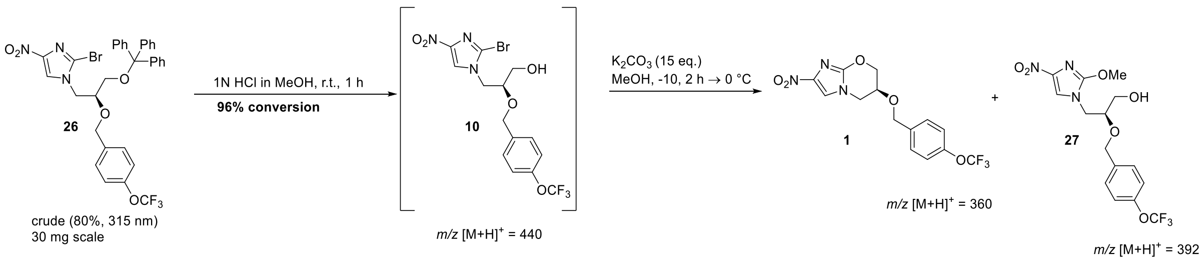

#	*T* [°C]	*t* [h]	area% (315 nm)				
			**27**	**10**	**1**	**26**	**BPs**
**1**	-10	2					
	0	20	4	23	44	-	29
	0	43	3	8	58	-	30
	0	67	6	4	63	-	27
	0	91	9	-	57	-	33

BPs = byproducs

In the following, the experiment was performed on gram scale:

Crude **26** (3.53 g) was dissolved in dry MeOH under nitrogen atmosphere. 3N HCl in MeOH (1.3 mL) was added and the mixture stirred at room temperature. for 3 h (99% conversion to **10** detected by LC-MS). The solution was cooled to –10 °C using a cryostat before K_2CO_3 (7.83 g) was added in a single portion. The mixture was stirred for one hour at –10 °C, then, the cryostat temperature was changed to 0 °C. After 38 h (complete conversion of **10** detected by LC-MS), H_2O (70 mL) was added and the mixture stirred at room temperature overnight. The precipitate was suction filtered and washed with five portions of water (5 mL each). After drying on the air overnight, the beige precipitate was dried in a desiccator for three days. The precipitate was suspended in MTBE (10 mL), heated to reflux for two minutes and stirred to room temperature. After filtration and washing with MTBE, the solid was air-dried first, followed by drying in vacuo at 40 °C. Pretomanid **1** (0.63 g, 1.8 mmol, 36% (related to 4.95 mmol **7**) was obtained as a colorless solid in 99.9% purity (HPLC, 315 nm).

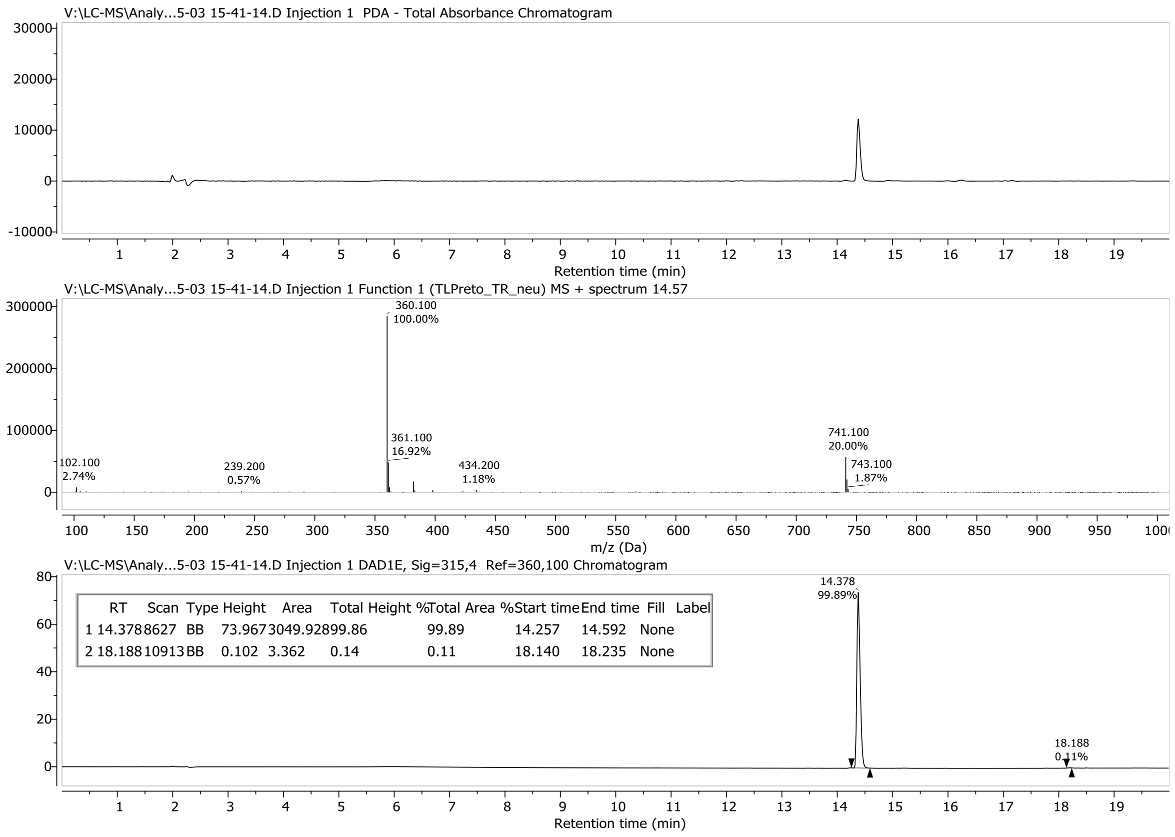

Figure S5. HPLC chromatogram (column: ACE C18 PFP, gradient MeCN:H2O (+ 0.1% formic acid): 5:95 (2.5 min), 5:95 to 95:5 (15 min), 95:5 (2.5 min), λ = 315 nm) of pretomanid **1** prepared through the Trityl-route.

Spectra:

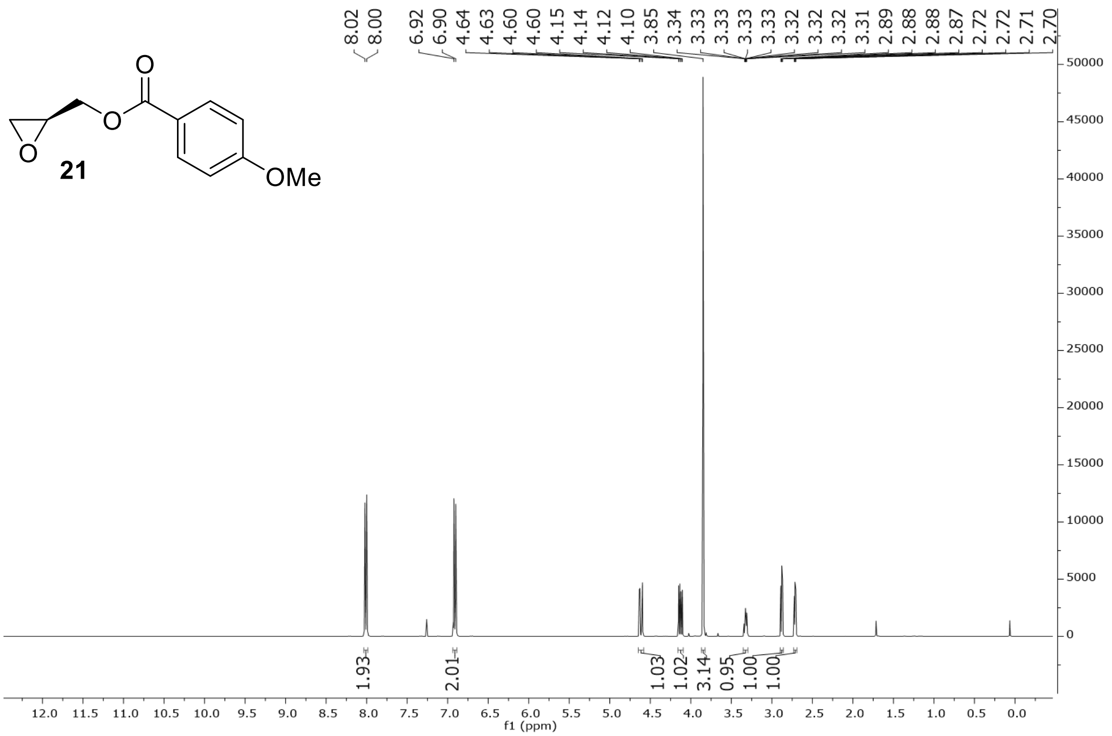

^{1}**H-NMR**-Spectrum (300 MHz, $CDCl_3$) of (*S*)-oxirane-2-ylmethyl 4-methoxybenzoate (**16**).

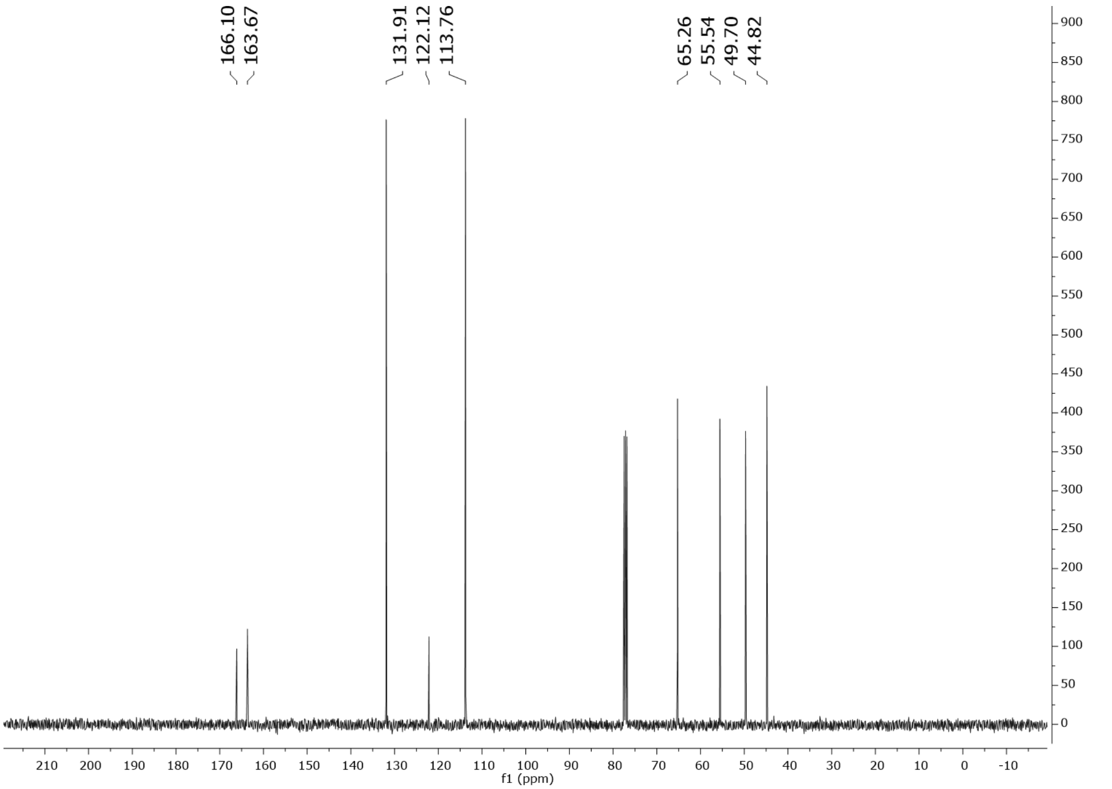

^{13}**C-NMR**-Spectrum (75 MHz, $CDCl_3$) of (*S*)-oxirane-2-ylmethyl 4-methoxybenzoate (**16**).

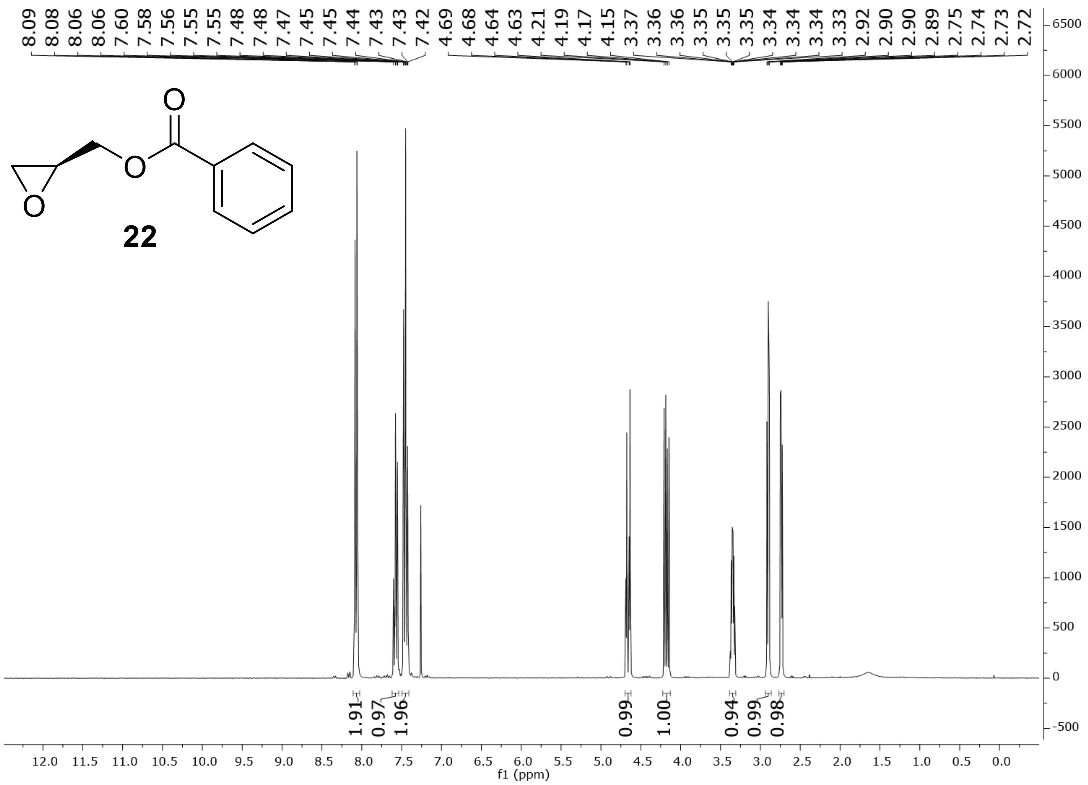

^{1}H-NMR-Spectrum (300 MHz, $CDCl_3$) of (*S*)-oxirane-2-ylmethyl benzoate (**17**).

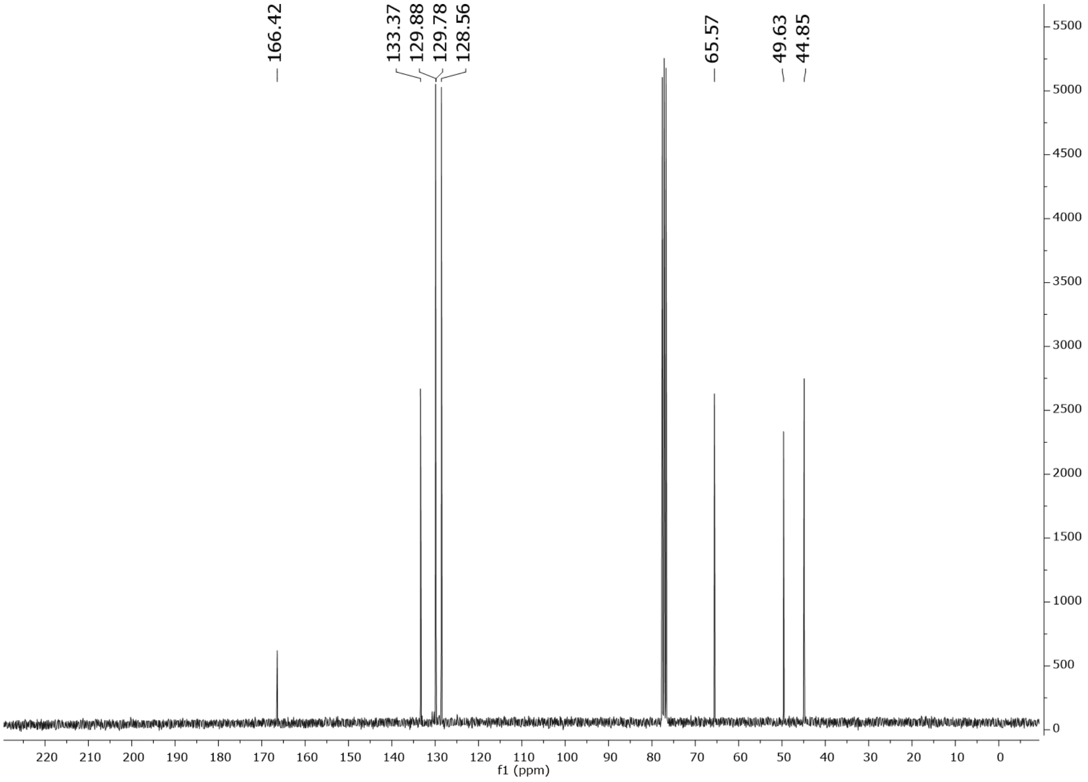

^{13}C-NMR-Spectrum (75 MHz, $CDCl_3$) of (*S*)-oxirane-2-ylmethyl benzoate (**17**).

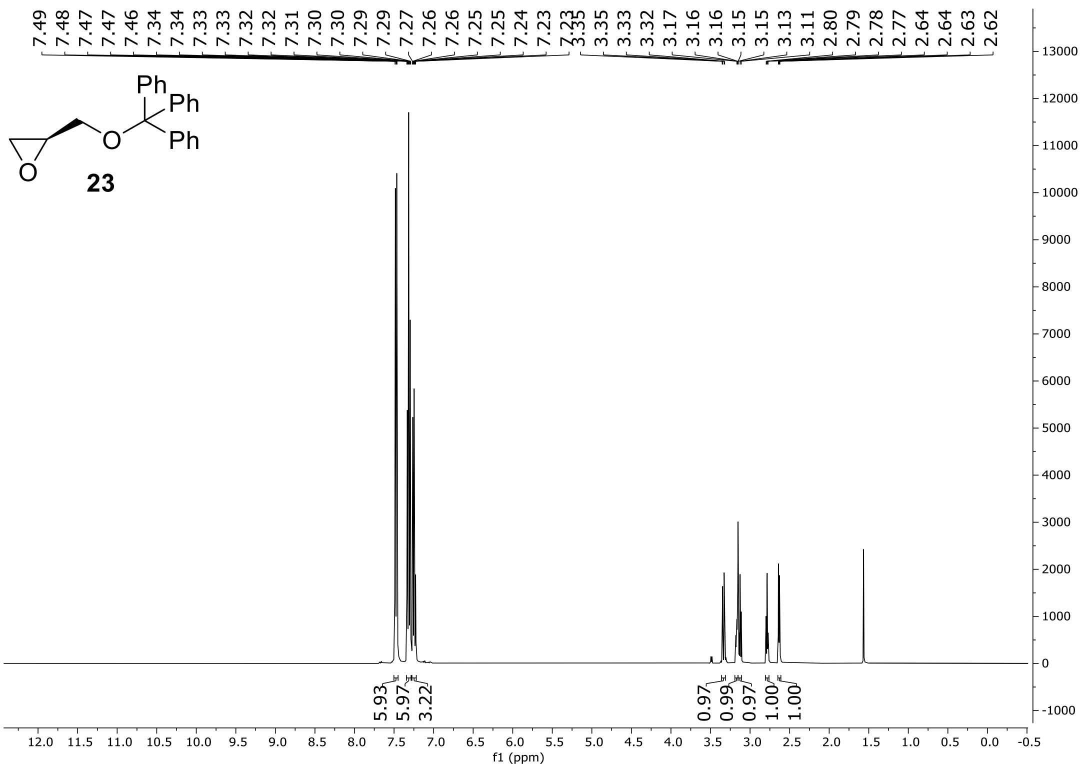

^{1}**H-NMR**-Spectrum (300 MHz, $CDCl_3$) of (*S*)-2-((trityloxy)methyl)oxirane (**18**).

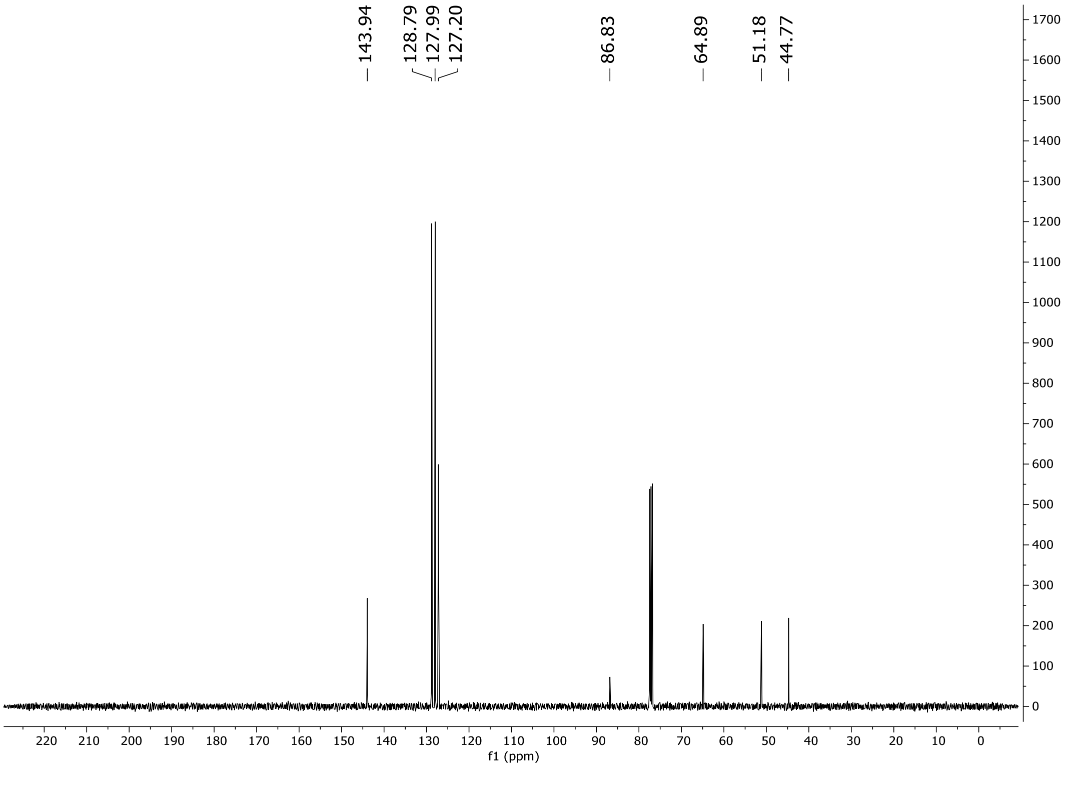

^{13}**C-NMR**-Spectrum (75 MHz, $CDCl_3$) of (*S*)-2-((trityloxy)methyl)oxirane (**18**).

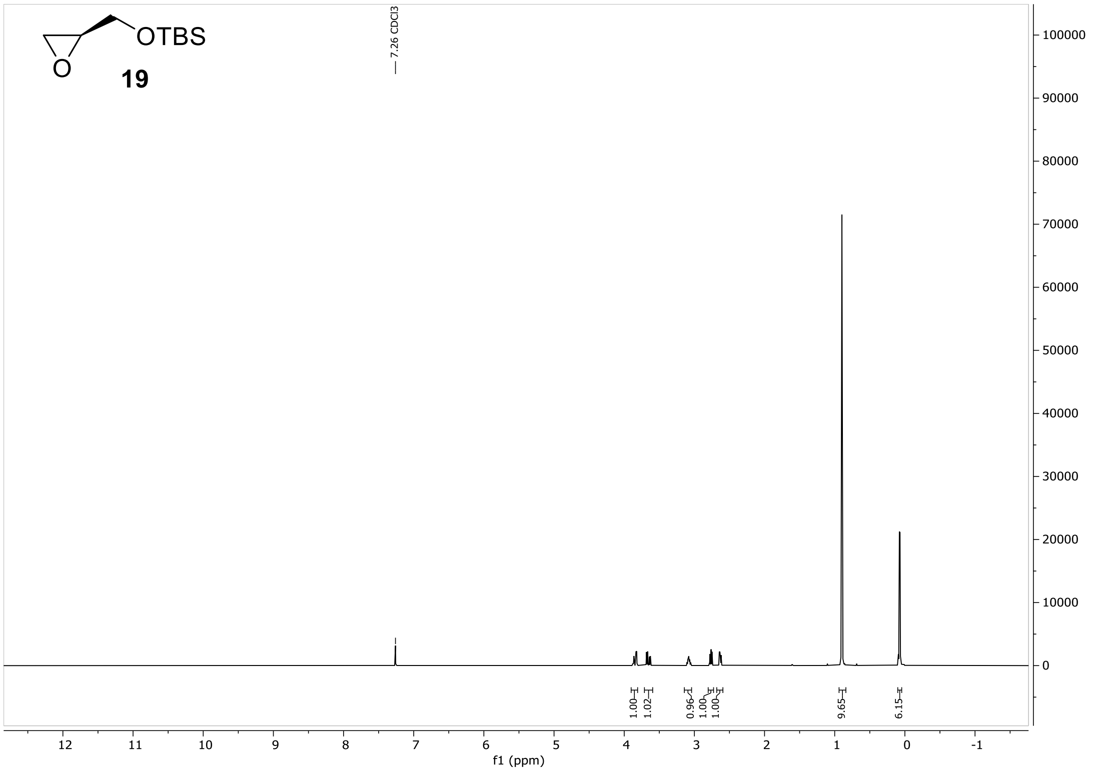

[1]**H-NMR**-Spectrum (300 MHz, $CDCl_3$) of (*S*)-*tert*-butyldimethyl(oxiran-2-ylmethoxy)silane (**19**).

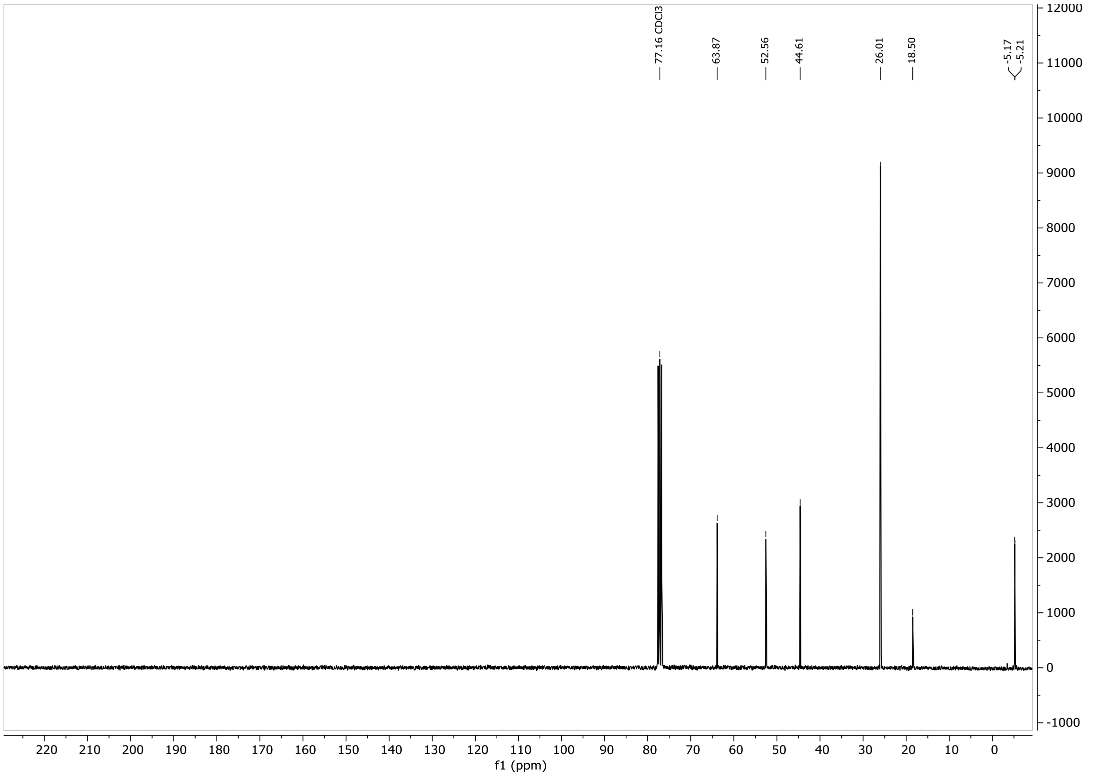

[13]**C-NMR**-Spectrum (75 MHz, $CDCl_3$) of (*S*)-*tert*-butyldimethyl(oxiran-2-ylmethoxy)silane (**19**).

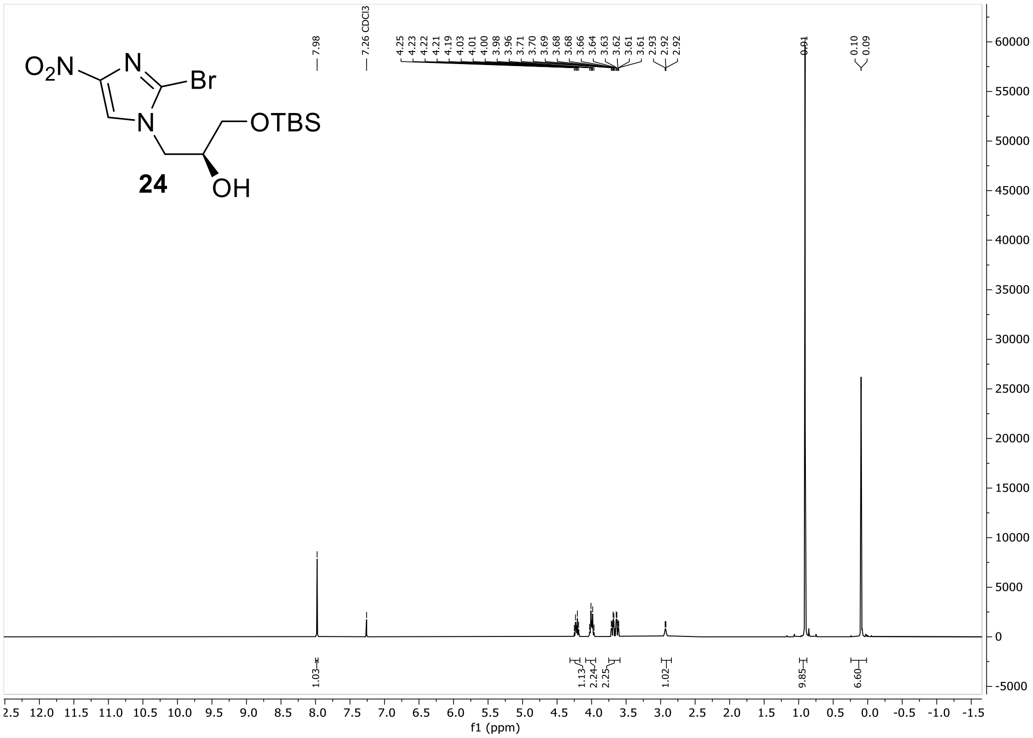

^{1}H-NMR-Spectrum (300 MHz, $CDCl_3$): (*S*)-1-(2-bromo-4-nitro-1*H*-imidazol-1-yl)-3-((*tert*-butyldimethylsilyl)oxy)propan-2-ol (**24**).

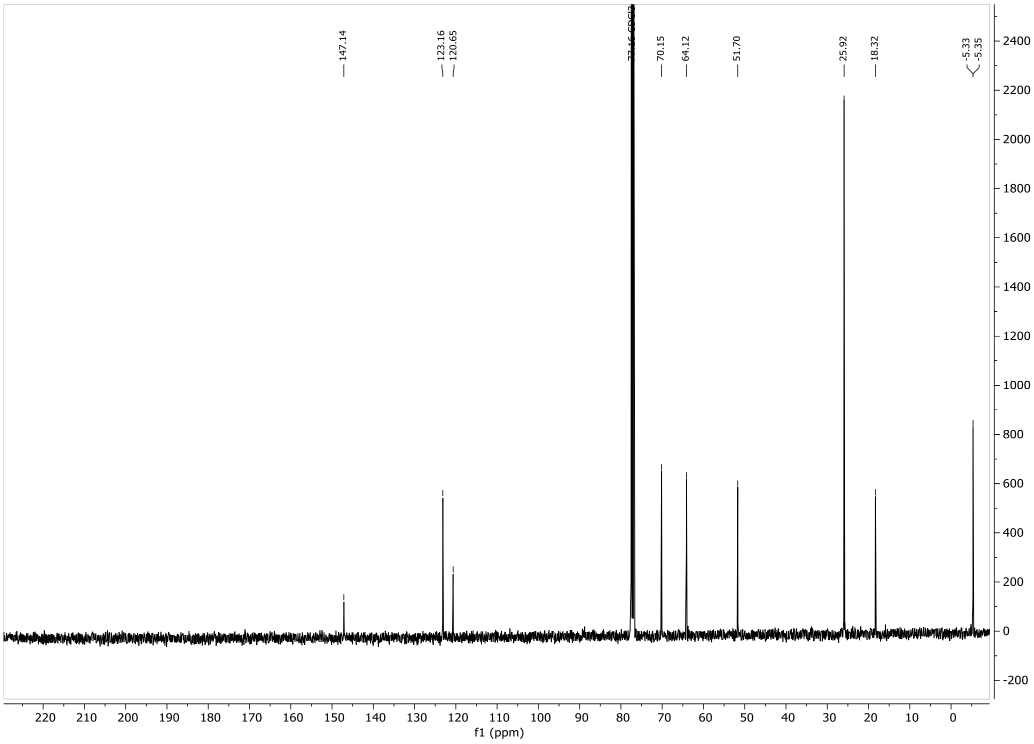

^{13}C-NMR-Spectrum (75 MHz, $CDCl_3$): (*S*)-1-(2-bromo-4-nitro-1*H*-imidazol-1-yl)-3-((*tert*-butyldimethylsilyl)oxy)propan-2-ol (**24**).

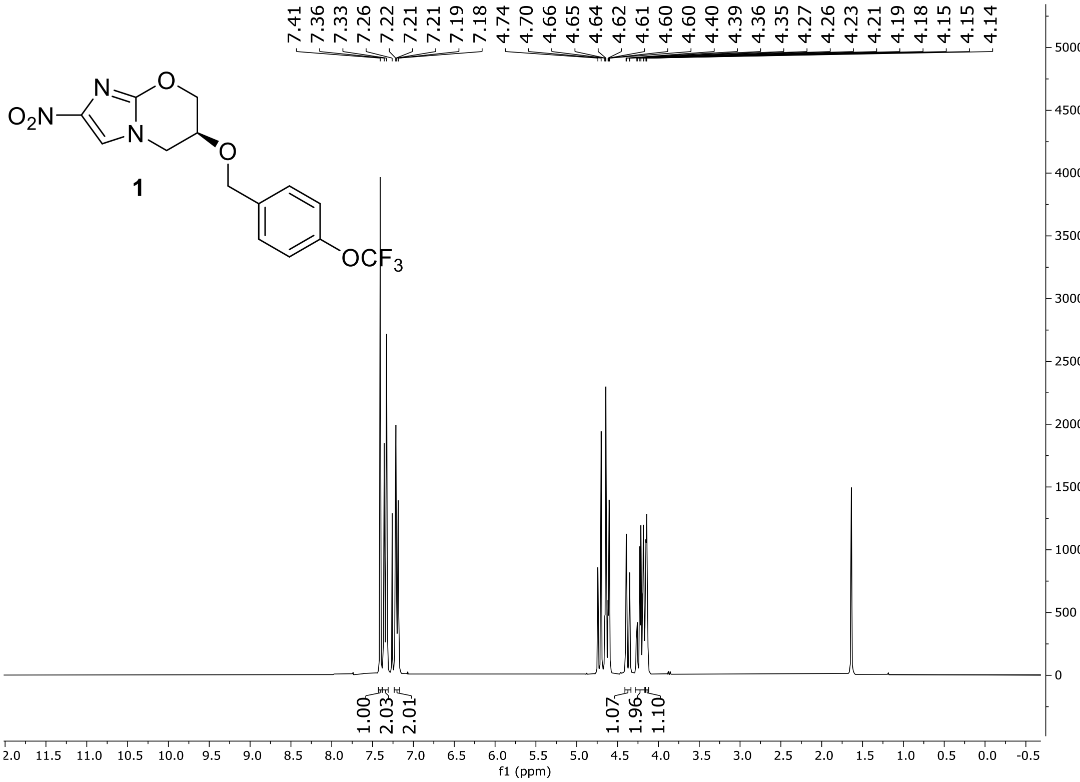

^{1}H-NMR-Spectrum (300 MHz, $CDCl_3$) of pretomanid **1**.

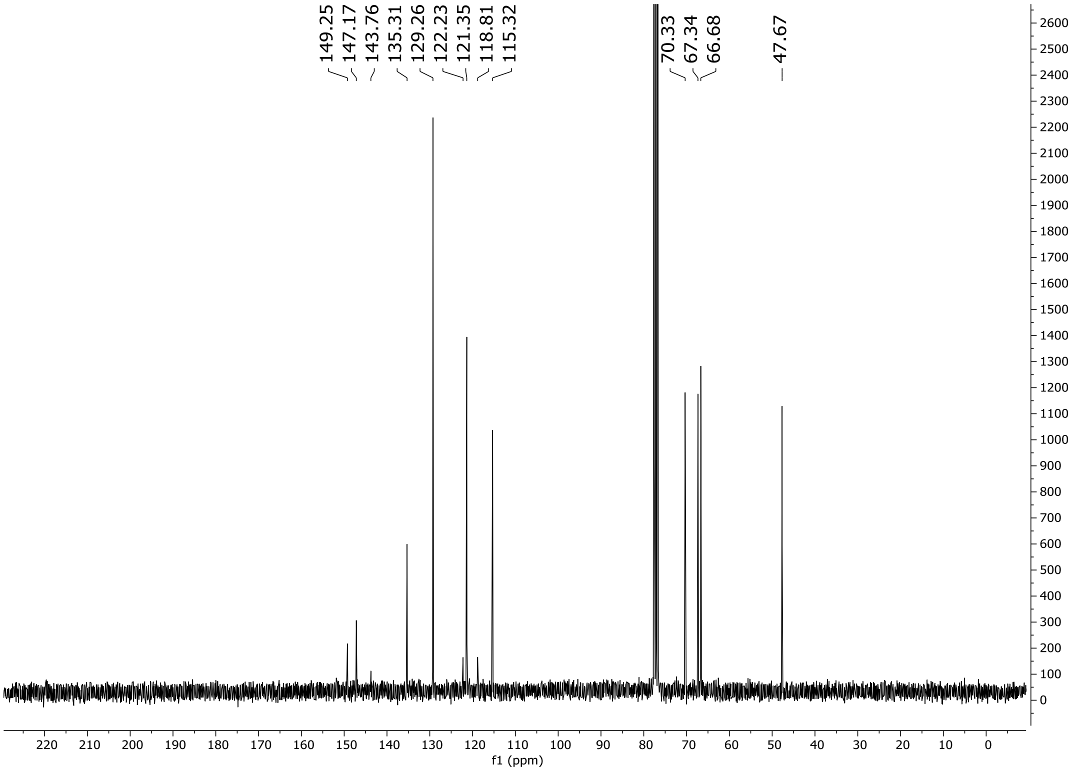

^{13}C-NMR-Spectrum (75 MHz, $CDCl_3$) of pretomanid **1**.

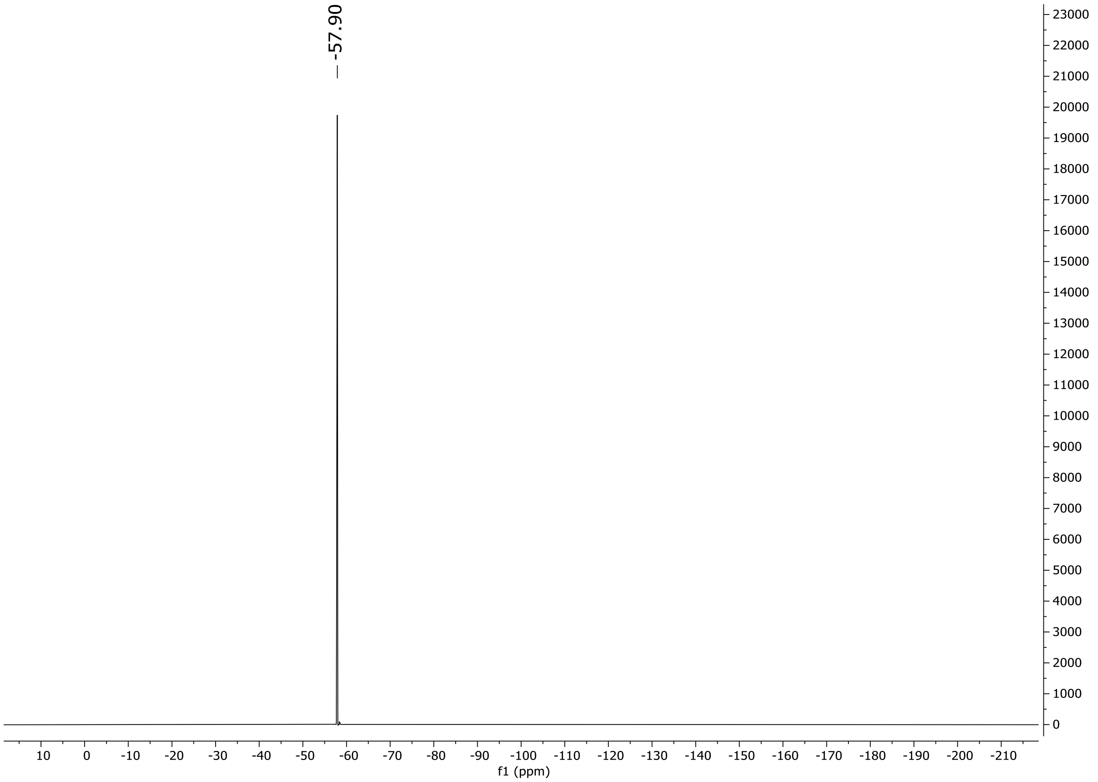

^{19}F-NMR-Spectrum (376 MHz, $CDCl_3$) of pretomanid **1**.
